# A Computational Approach Identifies Immunogenic Features of Prognosis in Human Cancers

**DOI:** 10.3389/fimmu.2018.03017

**Published:** 2018-12-21

**Authors:** Malini Manoharan, Nitin Mandloi, Sushri Priyadarshini, Ashwini Patil, Rohit Gupta, Laxman Iyer, Ravi Gupta, Amitabha Chaudhuri

**Affiliations:** ^1^MedGenome Labs Ltd., Bangalore, India; ^2^MedGenome Inc., Foster City, CA, United States

**Keywords:** tumor microenvironment, gene signatures, inflamed tumors, CD8^+^ T cell infiltration, TCR signaling, anergic and exhausted T cells and prognosis

## Abstract

A large number of tumor intrinsic and extrinsic factors determine long-term survival in human cancers. In this study, we stratified 9120 tumors from 33 cancers with respect to their immune cell content and identified immunogenomic features associated with long-term survival. Our analysis demonstrates that tumors infiltrated by CD8^+^ T cells expressing higher levels of activation marker (PD1^hi^) along with TCR signaling genes and cytolytic T cell markers (IL2^hi^/TNF-α^hi^/IFN-γ^hi^/GZMA-B^hi^) extend survival, whereas survival benefit was absent for tumors infiltrated by anergic and hyperexhausted CD8^+^ T cells characterized by high expression of *CTLA-4, TIM3, LAG3*, and genes linked to PI3K signaling pathway. The computational approach of using robust and highly specific gene expression signatures to deconvolute the tumor microenvironment has important clinical applications, such as selecting patients who will benefit from checkpoint inhibitor treatment.

## Introduction

Tumor microenvironment (TME) ecosystem, with its complex mixture of non-malignant and malignant cells, regulates tumor growth, progression to metastasis, treatment response, and development of resistance to therapy (1, 2). The TME ecosystem has come to the forefront of scientific scrutiny as a critical determinant of response to cancer immunotherapy drugs (3). Therefore, it has become imperative to develop methods and tools to examine the TME ecosystem in a greater detail and discover features that are permissive for mounting an anti-tumor immune response (4). The functional heterogeneity of the TME is contributed not only by the composition of different cell types in the tumor, but also their activation state, regulated by a complex milieu of chemical signals to which these cells are exposed. A further layer of complexity is introduced by the genetic changes that happen during tumor evolution (5). Delineating the hidden features within the TME is, therefore, a promising area for discovering predictive and prognostic biomarkers (4).

Two major strategies are used to analyze the TME: (1) Deconvolution of the complete cellular composition of the TME, and (2) Assessing the enrichment of individual cell types present in the TME. Deconvolution approaches are fraught with many different biases because of strict dependencies among all the inferred cell types (5). Therefore, assessing the enrichment of individual cell types is a better approach, if robust cell type-specific signatures are available. The existing signatures derived from differential expression of genes (6, 7), correlation of immune-related genes (8), or quartile-based methods (9) results in a large number of genes (usually a few thousand), whose expression is not highly specific to a given cell type and therefore unsuitable for the analysis. Recent studies have leveraged single-cell sequencing as a powerful tool to analyze the tumor microenvironment at a higher resolution (10–12). However, loss of cell viability and inefficient capture of rare cells, in particular, limit its application in a clinical setting.

In this study, we have created minimal gene expression signature profiles (MGESPs) for eight broad categories of immune cells (CD8^+^, CD4^+^ T cells, B cells, Treg cells, NK cells, Macrophages, Monocytes, and Neutrophils). Validation of the MGESPs on pure immune cell expression data generated by different platform technologies revealed a high degree of specificity in discriminating cognate from non-cognate cells. We demonstrate the utility of the MGESPs by enumerating the presence of different immune cell types in over 9,000 complex tumor microenvironments across 33 cancers. The analysis revealed different patterns of immune infiltration in different cancers driven by restricted expression of chemoattractant genes and by pathways dysregulated as a result of mutations in oncogenes and tumor suppressor genes. Additionally, we identified CD8^+^ T cell-specific markers whose expression strongly correlates with protective CD8^+^ T cell immunity in a pan-cancer setting. We have validated the utility of these marker genes by estimating a T-cell activation score on a independent cohort of patients with advanced melanoma treated with immunotherapy drugs. We observed that these genes can be used to monitor immunological response in patients.

## Results

### Minimal Gene Expression Signature Profiles (MGESPs) of Immune Cells

**Figure 1:**
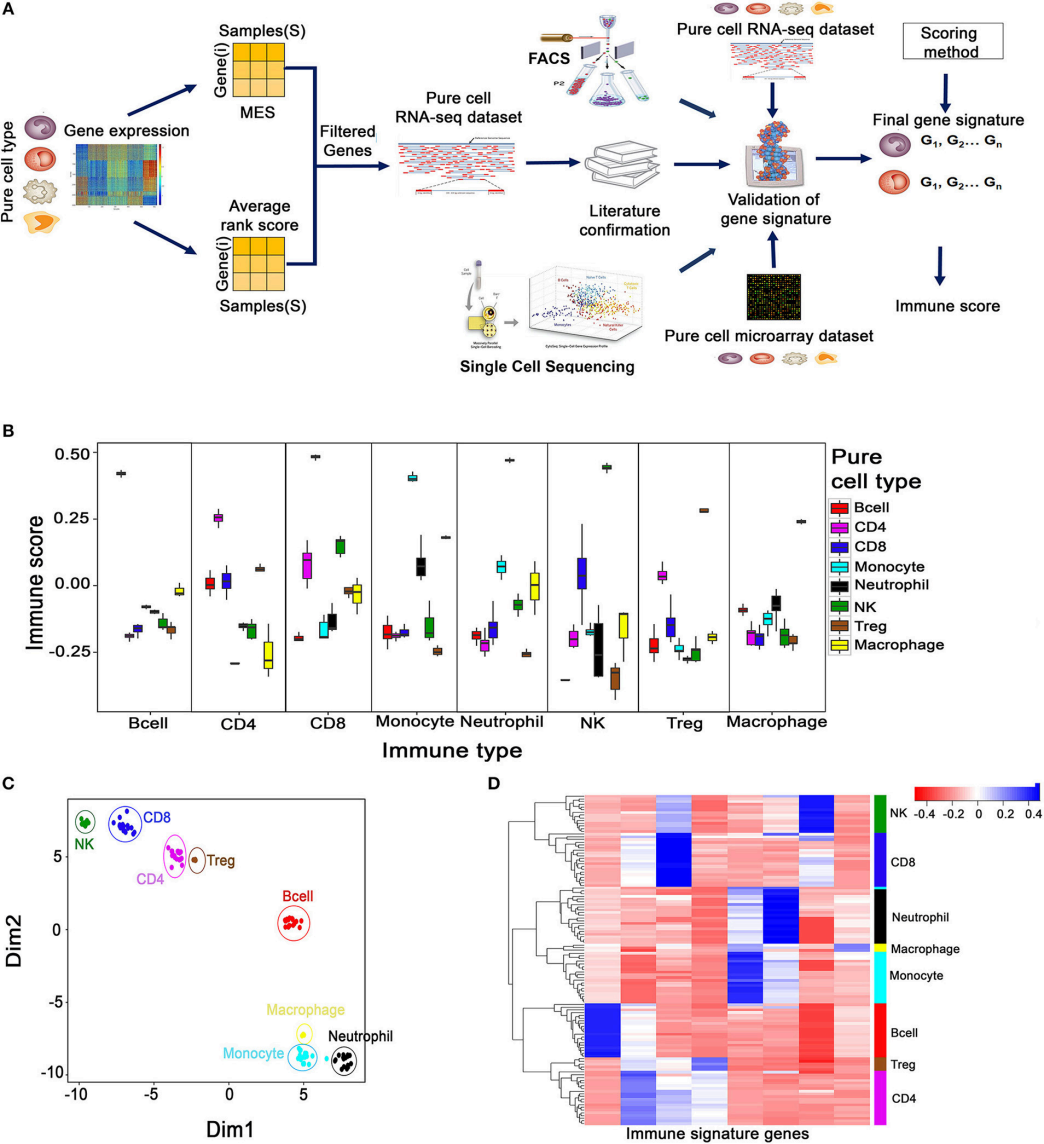
Creation and validation of minimal gene expression signature profile (MGESP) for eight different immune cells. **(A)** Workflow for creating and validating the MGESPs. (**B)** Validation of MGESPs on RNA-Seq data represented as boxplots with signature on the x-axis and immune score on the y-axis. Each facet represents the immune score calculated for the signature mentioned on the x-axis for both their cognate and non-cognate cell types. A higher score was obtained for the cognate cell types as represented in each facet (Wilcoxon Test : *P*-value < 0.05). **(C)** Visualization of immune cell-types using the expression of MGESPs on two-dimensional coordinates from t-stochastic neighbor embedding (t-SNE) algorithm. **(D)** Hierarchical clustering of immune cell-types on RNA-Seq data from pure immune cells.

We employed a three-step strategy to create non-overlapping cell type-specific gene signatures for eight immune cells—CD8^+^, CD4^+^, T cells, B cells, Treg cells, NK cells, Macrophages, Monocytes, Neutrophils, which we refer to as the MGESPs (Minimal Gene Expression Signature Profiles). The systematic approach used for creating the MGESPs is described in Figure 1A. In the first step, we used the ARS (Average Rank score) and the MES (Marker evaluation score) derived from large microarray datasets of pure immune cells as described in Wang et al. (13) to prioritize the genes. The ARS score was used to identify genes that are highly expressed in a given cell-type. We next used the MES score which is a measure of the intercellular variability to quantitatively assess the suitability of a gene to be a part of the MGESPs. In the second step, we verified the expression of the selected genes in pure RNA-Seq data to ensure that the signature genes for a given cell type show significant expression across different platforms. In the final step, the function of the prioritized genes was evaluated in the cognate cell type using literature curation. Next, we used the single sample Gene Set Enrichment analysis GSEA (ssGSEA) to derive a normalized score for each of the cell types in a given sample based on the MGESPs. ssGSEA uses an Empirical Cumulative Distribution Function (ECDF) of the genes in a given signature and the remaining genes to derive an enrichment score (14).

### Validation of Gene Signatures

We used pure immune cell expression data derived from four different platforms to validate our MGESPs (1) RNA-Seq data (122 samples) for all the eight immune cell types (15, 16); (2) microarray data (219 samples) across the eight cell types; (3) single cell expression data (2,764 single cells) covering B cells, Macrophages and NK cells (10); (4) FACS sorted immune cells from peripheral blood mononuclear cells (PBMCs) from the ImmPort, Study id :SDY305 (25 samples) (17) (Supplementary Table 1).

The robustness of the MGESPs was evaluated using the scores obtained for the eight-immune cell-types on pure cell RNA-Seq data as shown in Figure 1B. As shown in each facet of Figure 1B, all the gene signatures exhibited a significantly higher score for their cognate cell type compared to the non-cognate cell types. The high specificity of our signatures was further reconfirmed in an independent pure immune cell type microarray data (Supplementary Figure 1A). Further, expression-based clustering of MGESPs on a mixture of RNA-seq data from 122 samples separated the immune cell populations according to their developmental lineage as shown by the t-SNE plot (Figure 1C) (18). Figure 1D shows B-cells distantly clustered from other cell types. CD8^+^ cells clustered closer to NK cells and CD4^+^ cells clustered closer to Treg cells, respectively. Neutrophils, Monocytes, and Macrophages clustered closer to each other according to their developmental lineage. Hierarchical clustering of expression data from both RNA-Seq (Figure 1D) and microarrays (Supplementary Figure 1B) separated the immune cells from each other further reinforcing the robustness of the MGESPs. Next, we tested our signatures on single-cell sequencing data from tumor-infiltrated lymphocytes (B-cell, Macrophages, CD8^+^ T cells, and NK cells) in melanoma patients (GSE72056) (10) and observed higher scores for the cognate cell types (Supplementary Figure 1C). To further validate our signatures, we used FACS-sorted immune cells and compared signature-based prediction with FACS-based prediction of specific immune cell types (17). Cell-type-specific scores were calculated from RNA-seq data and correlated with the FACS data (Supplementary Figure 1D). Overall, our signatures correlated well with FACS data except for Treg cells where our signature identified the FACS sorted cells as CD4^+^ T cells. This could be because the markers used for identifying and sorting the Treg cells in this study did not include Foxp3, but used CD25, which is also expressed by non Treg cells. To further establish that the CD4 and the Treg signatures are indeed specific, we used data from 1,185 single cells labeled as T cells (GSE72056) (10) and applied CD4, CD8, and Treg signatures. The signatures separated the CD4 and Treg cells demonstrating the specificity of our signatures (Supplementary Figure 1E) Taken together, extensive validation of the MGESPs enabled detection of specific immune cell types with high specificity from complex transcriptomic data.

### The Immune Landscape of Human Cancers

**Figure 2:**
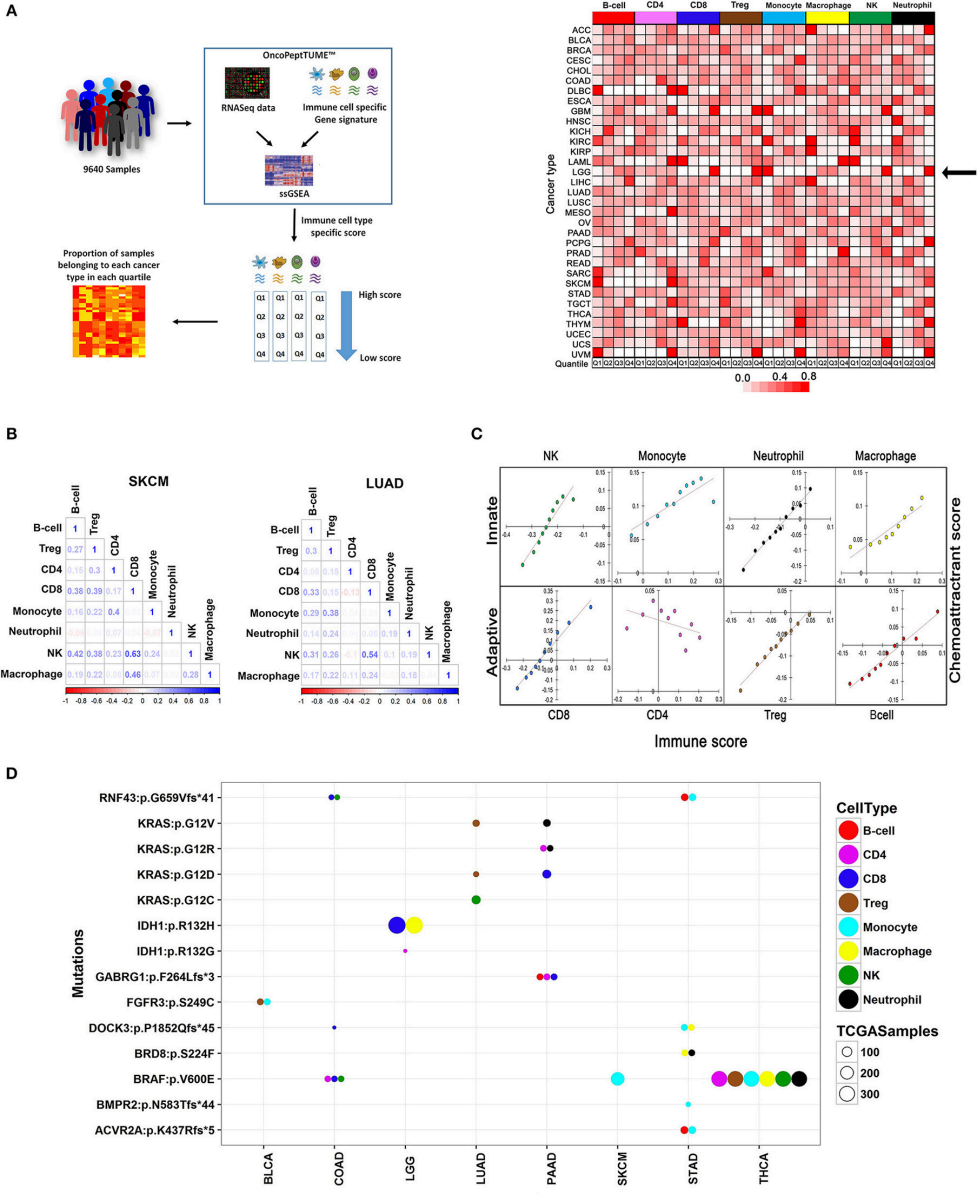
Comprehensive analysis of the immune landscape of 9,640 tumors across 33 cancers using MGESPs. **(A)** Workflow to identify cancers with the highest infiltration of a given immune cell-type (left panel). MGESP-derived score for each immune cell-type was calculated for each of the tumors in the dataset and arranged into quartiles. The number of samples in each quartile was used to create the heatmap (right panel). The color represents the proportion of tumor samples belonging to each cancer present in the quartile. Red and white color indicates a higher and lower number of tumor samples, respectively in a given quartile. **(B)** Represents the correlation of immune infiltration seen in SKCM and LUAD cancers. **(C)** Infiltration of immune cells is dependent on the expression of chemoattractant genes specific to each immune cell-type. Dependence is shown as a correlation plot of scores derived using a chemoattractant gene signature for each immune cell type and MGESP scores for each of the eight-immune cell-types across all cancers. **(D)** Enrichment of specific immune cells in tumors carrying mutations in a subset of oncogenes and tumor suppressor genes. Bubble plot showing genetic alterations showing significant correlation with the infiltration of different immune cells (*T*-test, *p*-value ≤ 0.005). Each colored bubble represents a specific immune cell, and the size of the bubble represents number of tumors carrying a specific mutation. See Supplementary Table 3 for a complete list.

We leveraged the whole transcriptome data from 9,640 tumors across 33 cancers and estimated the level of tumor-infiltrating immune cells using the MGESPs. We observed variable abundance of different immune cells in the tumor microenvironment (Supplementary Figure 2). To study the distribution of the infiltrating immune cells across the different cancer types and investigate the mechanisms of immune cell infiltration, we sorted the tumors by their immune infiltration scores and calculated the percentage of samples from each cancer present in each of the four quartiles (2,410 tumors in each quartile from all 33 cancers; Figure 2A, left panel). Next, we analyzed what fraction of tumor samples from different cancers is present in each quartile and plotted the % as a heatmap (Figure 2A, right panel). By focusing primarily on the first (Q1: Highest infiltration) and last quartile (Q4: Lowest infiltration), we were able to identify the pattern of immune infiltration in all cancers.

The first quartile of B cell infiltration (highest infiltration) was populated by DLBCL (Diffuse large B-cell lymphoma) cancer as expected (Figure 2A, right panel) and SKCM (skin cutaneous melanoma), UVM (uveal melanoma) and SARC (sarcoma). More than 95% of the samples from GBM (glioblastoma) and LGG (low-grade glioma) showed high infiltration of monocytes and high infiltration of NK and CD8^+^ T cells was detected in 98% of LAML (Acute Myeloid Leukemia) and 95% of THYM (Thymoma). An interesting finding of this analysis is that different renal cancer subtypes showed contrasting immune infiltration patterns. Kidney renal cell carcinoma (KIRC) being an immune-sensitive tumor (19) had a high infiltration of all the immune cell types except Treg cells (Figure 2A, right panel), dark red Q1 boxes, whereas kidney renal papillary cell carcinoma (KIRP) showed lesser infiltration of most immune cell types (Figure 2A, right panel), white Q1 boxes. Interestingly, kidney chromophobe cancer (KICH) showed a high infiltration of NK cells and low infiltration of other cell types, previously reported by immunohistochemistry analysis (20). Taken together, our analysis reveals both known and novel aspects of tissue-specific immune cell compositions in different cancers.

Immune cells interact with each other and with non-immune cells to define the inflammatory properties of the tissue microenvironment. For example, the co-occurrence of T cells and NK cells in tumors enhances the efficacy of cancer immunotherapy drugs (21). There has been no systematic analysis of co-infiltration of multiple immune cells across different cancers. Therefore, we used the TCGA data to investigate the landscape of co-infiltrating immune cells in all 33 cancers and observed interesting correlations (Supplementary Figure 3). For cancers that have shown a good response to immune checkpoint inhibitors (SKCM, KIRC, BLCA, LUAD, HNSC), a positive correlation between CD8^+^ T cells and NK cells, was observed with the strongest correlation detected in SKCM and LUAD (Figure 2B). We also observed good correlation between CD8^+^ T cells and NK cells in cancers where immunotherapy has not been tested yet, such as testicular germ cell cancer (TGCT), KIRP, and lung squamous cell carcinoma (LUSC) and speculate that a subset of tumor samples from these cancers may be sensitive to checkpoint inhibitor treatment. Co-infiltration of immune cells across different cancers is given in Supplementary Figure 3.

### Factors Regulating the Infiltration of Immune Cells in Tumors

To examine the mechanism of selective recruitment of specific immune cells in different tumors, we first considered the expression of the chemoattractant genes, which mobilizes immune cells to sites of inflammation as a possible mechanism. Analysis of co-expression of chemoattractant genes specific to each immune cell type obtained from literature survey (Supplementary Table 2) and their infiltration scores across all 33 cancers indicated that the chemoattractant gene expression scores were positively correlated with immune cell infiltration across all cancers, except CD4^+^ T-cells, where we observed an inverse correlation (Figure 2C).

Next, we considered mutations in oncogenes and tumor suppressor genes as possible drivers of immune cell infiltration through the expression of chemoattractant genes, or by other mechanisms, such as changes in the tumor stroma, or directly impeding the migratory behavior of immune cells. We selected tumor samples across different cancers enriched or depleted for different immune cells and analyzed mutations in all genes. We identified mutations in known and novel genes associated with enrichment or depletion of specific immune cells in different cancers (*T*-test: *P*-value < 0.05; Figure 2D and Supplementary Figure 4. We observed that the same mutation affected the infiltration of distinct immune cell populations in two different cancers. For example, in LUAD, higher Treg infiltration was seen in samples harboring oncogenic KRAS mutation (G12V/D), whereas the same mutation in pancreatic adenocarcinoma (PAAD) correlated with high infiltration of CD8^+^, CD4^+^ T cells, and Neutrophils. Several novel dependencies of clinical relevance were detected between mutations and infiltration of specific immune cells. For example, loss of function mutations in RNF43 and DOCK-3 genes were associated with higher infiltration of CD8^+^ T cells in colon adenocarcinoma (COAD; Figure 2D and Supplementary Figure 4A). RNF43 is a negative regulator of Wnt signaling (22), and DOCK-3 is a negative regulator of multiple signaling pathways including the Wnt β-catenin signaling (23). NK cell infiltration was higher in COAD samples carrying BRAF V600E and a frameshift mutation in RNF43 (Figure 2D, Supplementary Figure 4B). A snapshot showing the impact of other oncogenic mutations such as *KRAS, BRAF*, and *FGFR3* on the composition of the tumor-associated immune infiltrate is shown in Figure 2D and Supplementary Figures 4C–E. A complete list of mutations significantly associated with enrichment or depletion of immune cells is provided in Supplementary Table 3. We demonstrate that the relationship between driver mutations and their impact on immune infiltration is complex and is centrally dependent on the cancer type. Our analysis further reiterates that both chemoattractant gene expression and oncogenic mutations act together for the recruitment of specific immune cells in the TME and therefore contribute to the changes in the TME as the tumor develops over time.

### Prognostic Impact of Tumor-Infiltrated Immune Cells in Different Cancers

Cancer-related inflammation is the seventh hallmark of cancer (24, 25) and in many solid tumors higher levels of tumor-infiltrating leukocytes (TIL) is often correlated with increased progression-free survival (PFS) and overall survival (OS) (26–28). Both targeted, and large-scale genomic studies have revealed that different cancers benefit from infiltration of different immune cells. For example, CD8^+^ T cells, activated macrophages (M1-type), and NK cells are associated with good survival, whereas myeloid-derived suppressor cells (MDSCs), Treg cells and alternatively activated macrophages (M2-type) are associated with poor survival (29, 30).

**Figure 3:**
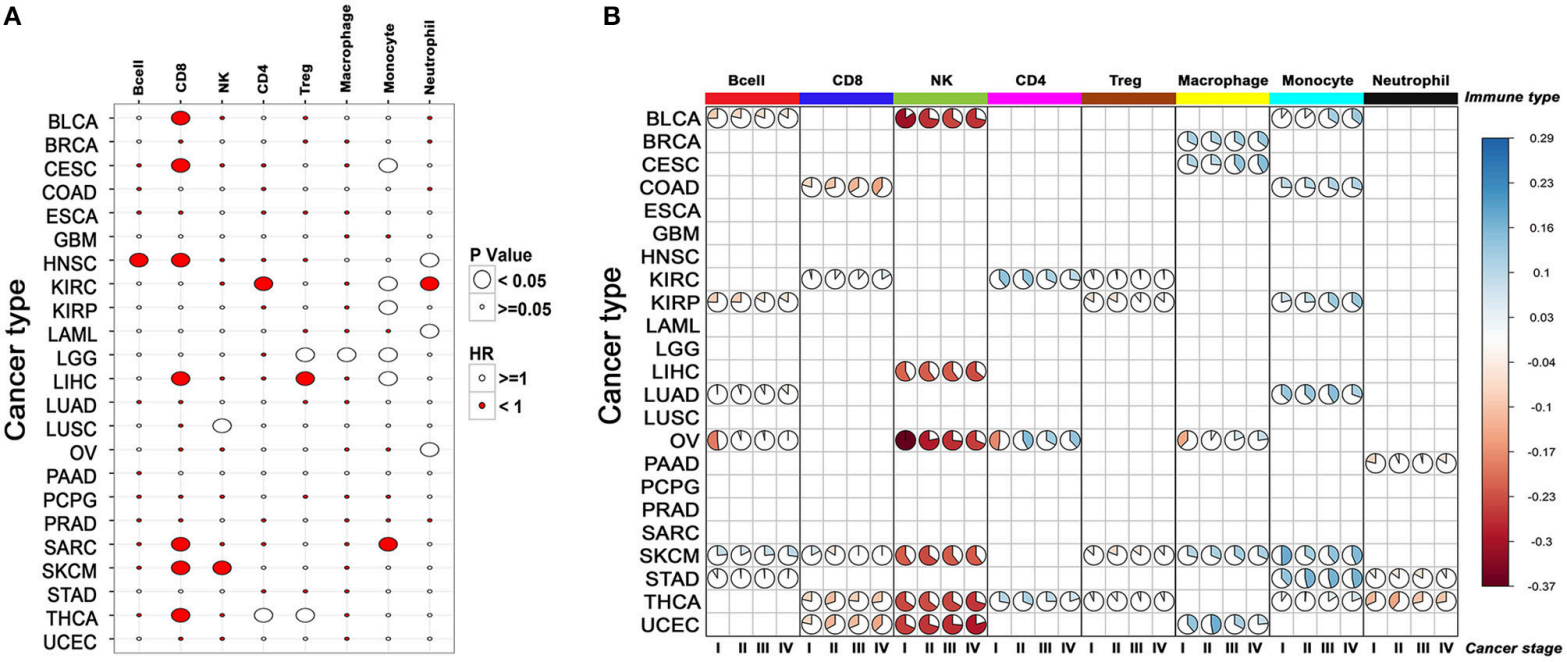
The relationship between the composition of immune infiltrate and its effect on patient survival across cancers. **(A)** Correlation between infiltration of different immune cells and patient survival. For each cancer, survival benefit between the top and bottom 20% tumor samples infiltrated by specific immune cells was compared. Size of the bubble shows significance (*p*-value < 0.05), red and white indicates good and poor prognosis, respectively. **(B)** Changes in the composition of immune infiltrate with tumor stage in different cancers. Only the immune scores differing significantly between cancer stages for a given cell-type are represented by the pie plot (cor. test, *p*-value < 0.05).

In accordance with other published studies, CD8^+^ T cell infiltration was associated with improved survival (See section Materials and Methods) in seven of the 23 cancers (31–36), whereas monocyte/macrophage infiltration exhibited poor survival in seven of the 23 cancers (Figure 3A, Supplementary Table 4). Both CD8^+^ T cells and NK cells showed a good survival benefit in SKCM samples and interestingly as described earlier, infiltration of CD8^+^ and NK cells were also highly correlated in this cancer. In addition to CD8^+^ T cell infiltration, infiltration of B cells also showed a good survival benefit in HNSC. Increased numbers of intraepithelial CD8^+^ T cells in metastatic tumors, as well as large numbers of peritumoral B cells in lymph node metastases, have been shown to be associated with favorable outcome in previous studies (37).

To further investigate whether the immune cell composition of the tumor changed from being protective to permissive as cancer progressed, we mapped the relative levels of immune cells in early and late-stage cancers. Our analysis indicated that in many cancers, such as COAD, SKCM, thyroid cancer (THCA), and uterine corpus endometrial carcinoma (UCEC) there was a progressive decrease in CD8^+^ T cell infiltration with increased disease stage (Figure 3B). Conversely, monocyte infiltration increased with stage in many cancers, indicating adverse impact on survival.

### CD8^+^ T Cell-Dependent Long-Term Survival Benefit in Human Cancers

**Figure 4:**
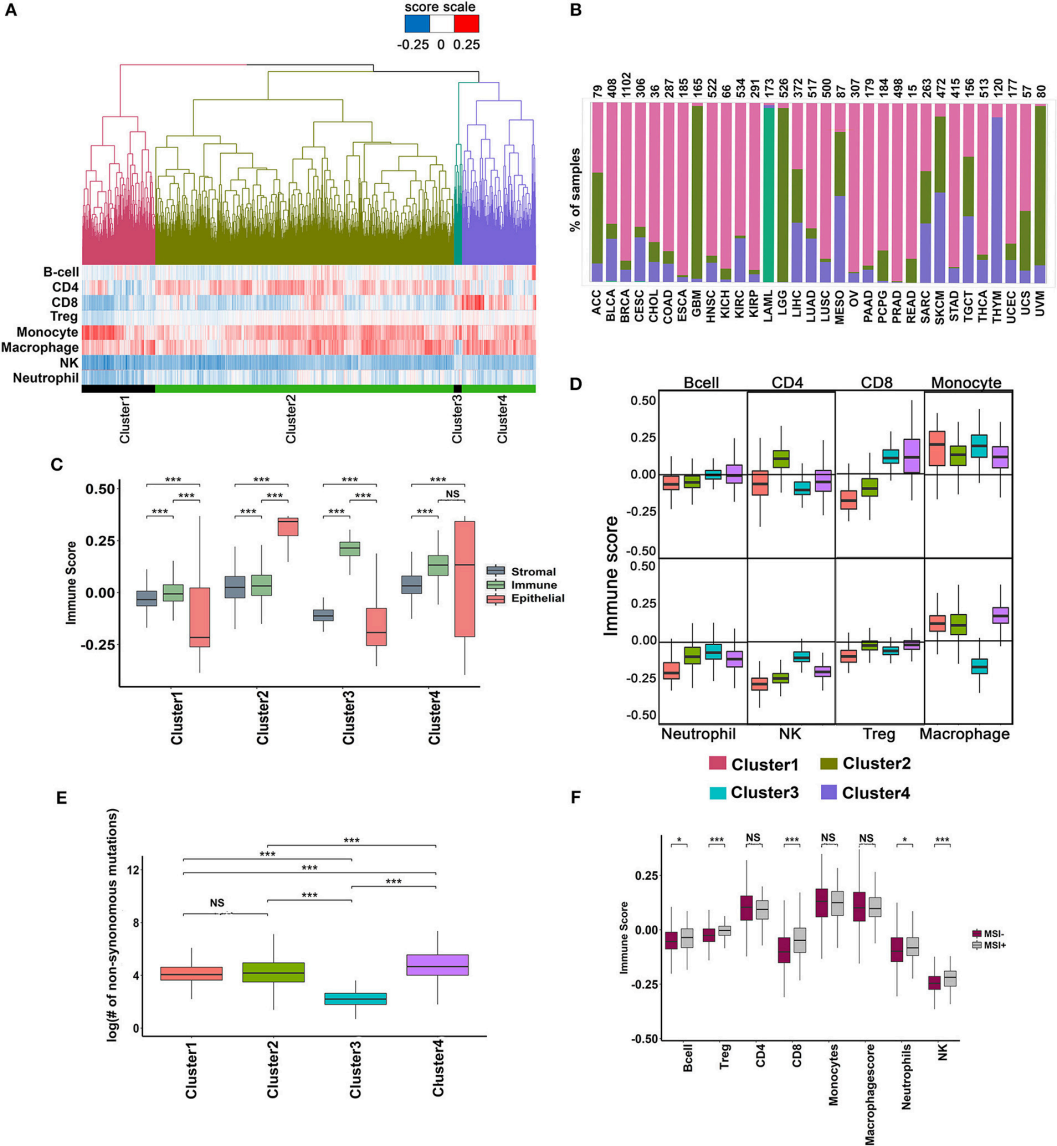
Analysis of immune infiltrate of TCGA tumors using minimal gene expression signature profiles. **(A)** Unsupervised clustering of 9,548 TCGA tumors based on the infiltration of eight different immune cells. Four major clusters are shown with their corresponding immune cell infiltration pattern represented as a heatmap below the dendrogram. **(B)** Percentage of tumors in each cluster distributed across different cancers. **(C)** Distribution of Stromal, Immune and Epithelial content of samples in each cluster. The scores were derived using signatures from Yoshihara et al. (43). The significance was ascertained by Wilcoxon text, *P*-value < 0.05. **(D)** Boxplot showing the variation in the distribution of immune infiltration scores for each immune cell type across the four clusters (Wilcoxon text, *P*-value < 0.05). **(E)** Boxplot showing variation in the distribution of the total number of mutations in the cluster (Wilcoxon text, *P*-value < 0.05). **(F)** Variation in the immune score distribution of the MSI+ and MSI- tumors (Wilcoxon text, *P*-value < 0.05).

A pro-inflammatory tumor microenvironment characterized by the presence of CD8^+^ T cells, NK cells, and M1-type macrophages is strongly correlated with long-term survival benefit, whereas an immune suppressive microenvironment infiltrated by Treg cells, MDSCs and alternatively activated macrophages (M2-type) predict poor survival (38, 39). There has been a renewed interest in defining the immunogenic state of a tumor to predict response to checkpoint blockade inhibitors. Analysis performed in the previous section suggested that prognosis was correlated with infiltration of specific immune cell-types. To investigate the mechanism of prognosis, we performed unsupervised clustering of 9,120 tumor samples across 33 cancers based on their combined immune infiltrate composition, instead of analyzing infiltration of one or few cell types as reported in other studies (40–42). The tumor samples clustered into four major groups according to the relative content of eight different immune cells (Figure 4A).

The distribution of different cancers in each of the clusters is shown in Figure 4B. Cluster-1 was enriched for uveal melanoma (UVM), low-grade glioblastoma (LGG), and glioblastoma (GBM; Figure 4B), whereas cluster-3 was exclusively composed of acute myeloid leukemia (LAML, 170 of 173 tumor samples). Clusters-2 and 4 were composed of a mix of different cancer types (Figure 4B). Cluster-1 and 3 had poor epithelial content, whereas cluster-2 and 4 were enriched in epithelial tumors (Figure 4C). The stromal content of cluster-2 and 4 was significantly high compared to cluster-1, and 3 and the immune content of cluster-3 and 4 were significantly higher than clusters 1 and 2 (Figure 4C). Analysis of the immune infiltrate compositions of cluster-1 and 2 revealed poor infiltration of CD8^+^ T cells and NK cells but rich in macrophages and monocytes (Figure 4D). Cluster-2 had significantly higher CD4^+^ T cells compared to all other clusters, whereas Cluster-3 and 4 were rich in CD8^+^ T cells (Figure 4D). As expected, cluster-3 containing exclusively of LAML (Acute Myeloid Leukemia) samples had significantly lower macrophage content than all other clusters (Figure 4D). The mutational burden was slightly higher in cluster-4 tumors (Figure 4E), which correlated with higher CD8^+^ T cell infiltration in cluster-4. Tumors with high mutation burden, such as MSI^+^ tumors showed higher CD8^+^ T cell infiltration. However, cluster-4 with high CD8^+^ T cell infiltration contains < 5% of all MSI^+^ tumors suggesting that other factors, besides mutation burden regulate CD8^+^ T cell infiltration (Figure 4F).

Given that each cluster is unique in its immune cell composition, we investigated differences in survival between the clusters. Cluster-4 tumors showed slightly better prognosis compared to cluster-1, or cluster-2, confirming that infiltration of CD8^+^ T cells is associated with long-term survival benefit (Supplementary Figures 5A,B). Of the 1,552 cases in cluster-4, 1,200 belongs to the alive group and the rest were deceased. We used these two groups of tumors to identify inflammatory features in CD8^+^ T cell-high tumors that were associated with long-term survival. The immune microenvironment profile of the alive group showed slightly higher content of CD4^+^ and CD8^+^ T cells compared to the deceased group. The alive group was also characterized by lower infiltration of Treg cells and monocytes compared to the deceased group (Supplementary Figure 5C). However, we failed to observe any differences in the expression of inflammation markers between the two groups, though the immune-suppressive markers showed lower expression in the alive group (Supplementary Figure 5D, Supplementary Table 5). Next, we examined the functional state of CD8^+^ T cells in the two groups. Significantly, while CD8^+^ T cells expressed the activation marker PD-1 in both alive and deceased groups, the deceased group was specifically enriched in CD8^+^ T cells expressing anergic and exhaustion markers—CTLA-4, LAG3, and TIM3 (Supplementary Figure 5E) indicative of the presence of dysfunctional CD8^+^ T cells in these group of tumors. In addition, the dysfunctional T cells in the deceased group showed reduced expression of CTL markers (Supplementary Figure 5F), further confirming their weak anti-tumor activity.

To further investigate the significance of CD8^+^ T cell function in determining prognosis, we analyzed the cancer types represented in cluster-4 and the proportion of alive and dead in each cancer type as shown in Supplementary Figure 6A. Tumors belonging to the deceased group were present in most cancer types except thyroid cancer where all tumors belonged to the alive group. Few cancers such as kidney renal carcinoma (KIRC), mesothelioma (MESO), skin cutaneous melanoma (SKCM), and ovarian (OV) contain >25% of dysfunctional CD8^+^ T cells higher than the rest of the cancers (15–20% dysfunctional T cells). This analysis confirmed that the unsupervised clustering method did not introduce a bias in the selection of cancer types that could have confounded our correlation between dysfunctional CD8^+^ T cells and poor survival. We also determined the mutation counts between tumors belonging to the alive and dead groups and found them to be similar (Supplementary Figure 6B). Next, we stratified the tumors according to their stage and determined the proportion of early (stage-1&2) or late (stage-3&4) tumors in the alive vs. the dead group (Supplementary Figure 6C). We observed an increase in the proportion of early-stage tumors in the alive group and late-stage tumors in the dead group suggesting that infiltration of functional and dysfunctional CD8^+^ T cells correlated with early or late-stage tumors, respectively.

**Figure 5:**
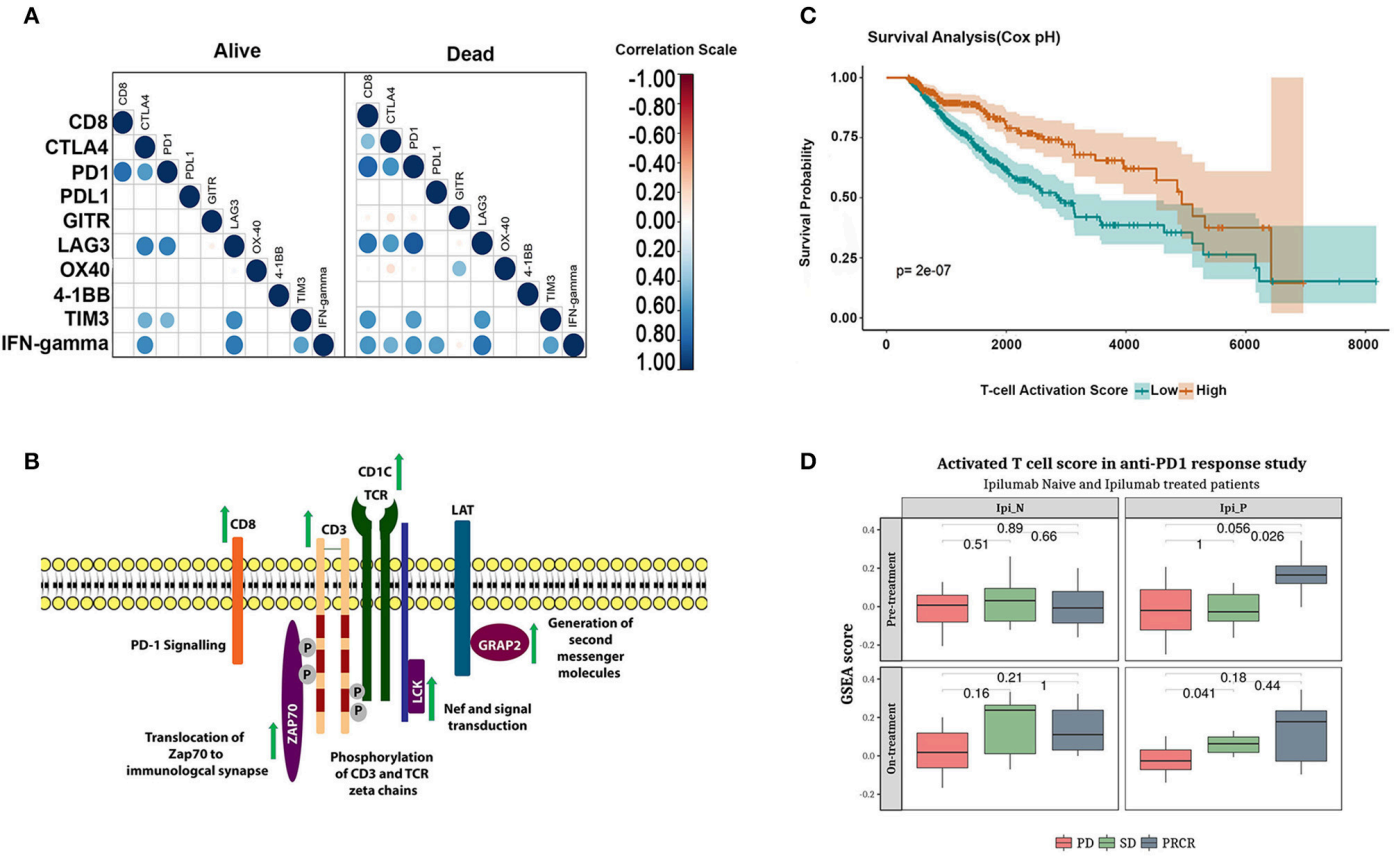
Functional phenotype of CD8^+^ T cells correlate with long-term survival and response to checkpoint inhibitor treatment. **(A)** correlation between CD8^+^ T cell infiltration and expression of anergic and exhaustion markers in early and late-stage tumors in alive and the deceased groups. **(B)** Schematic representation of the genes upregulated in the alive subjects of cluster-4. **(C)** Kaplan-Meier plot showing the survival difference associated with patients with high and low T-cell activation scores adjusted for age and stage using cox regression. **(D)** Boxplot showing enrichment of functional CD8^+^ T cell score in the melanoma cohort who responded to Ipilimumab or Nivolumab monotherapy (Student's *T*-test, *p*-values represented in the figure).

Surprisingly, a closer inspection of the functional status of CD8^+^ T cells in early-stage tumors revealed that whereas tumors in the alive group showed the exclusive signature of functional CD8^+^ T cells, the deceased group showed the presence of dysfunctional CD8^+^ T cell signature (Figure 5A). This observation strongly substantiates our hypothesis that the presence of functional CD8^+^ T cells is a hallmark of long-term survival for early-stage tumors. The late-stage tumors in both alive and dead groups contain dysfunctional CD8^+^ T cells, although expression of IFN-γ was restricted to the tumors belonging to the dead group (Figure 5A).

Having identified that the tumor microenvironment of the deceased group is enriched in dysfunctional CD8^+^ T cells, we examined factors that may have contributed to the anergic/exhausted phenotype. We analyzed genes differentially expressed between the two groups and mapped the differentially expressed genes onto pathways using REACTOME (44) (Supplementary Table 6). A list of differentially expressed genes is shown in Supplementary Table 7. We detected significant upregulation of a core network of genes functioning in T cell receptor (TCR) signaling specific to the alive group (Figure 5B and Supplementary Figure 7). Remarkably, all the upregulated genes encoded proteins of the TCR complex and proximal kinases that transduce TCR signaling following receptor activation (Figure 5B). We further assessed the contribution of these genes on patient survival using Cox regression analysis (see Materials and Methods section for deails of the analysis). Briefly, we computed activation scores as weighted average of gene expression of the genes associated with an activated phenotype of T-cells (Supplementary Figure 7). The survival analysis showed that high T-cell activation score was associated with an improved survival independent of age, stage and cancertype (*p*-value: 2e-07; Figure 5C). The association was verified using a multivariate cox regression analysis. The hazard ratios were 0.49 (95% CI 0.36–0.67) for the group with high T-cell activation scores (Supplementary Table 8).

These observations suggested that the dysfunctional state of CD8^+^ T cells in the deceased group was linked to the reduced expression of TCR signaling genes and higher expression of genes conferring an anergic and exhausted phenotype. This is the first systematic study to demonstrate key molecular mechanisms determining long-term survival using an immunogenomic approach.

A recent analysis of melanoma samples treated with nivolumab reported poor response to cancer immunotherapy drugs when their tumor microenvironment was infiltrated by dysfunctional T cells (45). The TCR signaling gene signature identified in this analysis was independently validated in the gene expression dataset from a cohort of patients with advanced melanoma (GSE91061). The change in enrichment of the T-cell activation score derived from the functional CD8^+^ T cell signature was applied to this cohort of patients. Patients who achieved partial or complete response to Ipilimumab (PR/CR) showed higher functional CD8^+^ T cell score (Figure 5D upper panel–right box) compared to Ipilimumab untreated patients. Further, patients who progressed on Ipilimumab but responded to Nivolumab (SD and PR/CR; Figure 5D lower panel, right box) showed higher functional CD8^+^ T cell activation score. In summary, the functional CD8^+^ T cell score was associated with response to both checkpoint inhibitors Ipilimumab and Nivolumab.

## Discussion

The immune checkpoint blockade therapies have achieved significant clinical success by targeting the T cell compartment in many cancers (46–48). However, only a small subset of patients achieves a long-term benefit from these therapies. Multiple studies have uncovered a correlation between clinical response to checkpoint blockade with pre-existing tumor-infiltrating lymphocytes, neo-antigen load, T-cell repertoire, reduced angiogenesis, and lack of mesenchymal cells in the tumor stroma (49–51). However, sensitive biomarkers to select patients in the clinic who will benefit from a checkpoint blockade therapy are not available. In this study, using a systematic immunogenomic approach we describe key molecular features that determine survival and identify novel biomarkers of inflamed tumors. Our success in stratifying tumors according to their immune cell infiltration was largely contributed by highly specific and robust MGESPs for eight different immune cell types. The published immune cell-specific gene expression signatures from multiple studies lack specificity and sensitivity for analyzing the complex tumor microenvironment.

We profiled the immune landscape of 9,640 tumors covering 33 cancer types using the MGESPs. Our analysis shows that tumors have distinct immune cell infiltration profiles that cannot be predicted apriori from analysis of tumor mutation burden or from mutations in oncogenes and tumor suppressor genes. We find that expression of immune cell-specific chemoattractant genes is strongly correlated with the infiltration of specific immune cells in all cancers (52, 53). In addition, our study reveals that associations between specific genetic alterations in oncogenes and tumor suppressor genes and the impact of such alterations on immune cell infiltration are cancer-specific. For example, a novel association was detected between mutations in two different modulators of the Wnt signaling pathway (RNF-43 and DOCK3) and increased CD8^+^ T cell infiltration in colorectal adenocarcinoma (COAD). RNF-43 is an E3 ligase that destabilizes frizzled receptors on the membrane (FZD) by ubiquitination and proteasome degradation resulting in the reduced surface expression of these receptors to induce down-regulation of the Wnt signaling pathway (54, 55). Dedicator of cytokinesis-3 (DOCK3) protein binds β-catenin and prevents its nuclear translocation thereby negatively regulating the Wnt-β-catenin signaling. We postulate that the frameshift mutations in RNF-43 and DOCK3 genes identified in the study are expected to increase Wnt signaling—the former by stabilizing the FZD receptors on the membrane (54) and the latter by releasing β-catenin for nuclear translocation (23). Our observation that increased infiltration of CD8^+^ T-cells correlated with loss of function mutations in RNF-43 and DOCK3 in human colon adenocarcinoma contrasts with the findings described in Spranger et al. (56). Further work is needed to delineate the complex role of Wnt signaling in immune cell infiltration. Our analysis also uncovered that BRAF V600E mutation in thyroid cancer (THCA) creates an immune suppressive tumor microenvironment by increasing Treg, neutrophil, and monocyte infiltration, while decreasing the infiltration of NK cells and CD4^+^ T-cells. This study stands out to be one of the largest analysis where the impact of all mutations across 33 cancers have been systematically associated with the enrichment or depletion of a variety of immune cells uncovering novel targets for immunopotentiation.

An interesting finding of our analysis, that tumor samples across different cancers cluster by their immune cell content supports the hypothesis that even when tumors originate from the same tissue-type, they become highly divergent as a result of their tumor microenvironment composition. A large proportion of TCGA tumors (~25%) are infiltrated with high CD8^+^ T cells (Figure 4A, cluster-4). Given that cancer immunotherapy drugs work better if tumors are infiltrated by CD8^+^ T cells, deeper analyses of the microenvironment of these tumors promise discovery of additional features of good prognosis and increased response to checkpoint blockade. Indeed, our analysis reveals that CD8^+^ T cells expressing higher levels of anergic and exhaustion markers, which are hallmarks of dysfunctional T-cells were enriched in the deceased group compared to the alive group (57). Further support to our hypothesis that CD8^+^ T cell phenotype contributed significantly to survival was shown by multivariate Cox regression analysis, where high T cell activation score correlated with long term survival. Significantly, this correlation was further substantiated by the finding that early stage tumors in the deceased group was enriched in dysfunctional CD8^+^ T cells, whereas tumors in the alive group showed the functional CD8^+^ T cell signature. The late stage tumors in both groups showed signature of dysfunctional CD8^+^ T cells, although the magnitude of dysfunctionality could not be assessed by our approach. However, given that IFN-γ transcript level correlated strongly with the dysfunctional phenotype of CD8^+^ T cells and was present only in the deceased group argues for further heterogeneity in the dysfunctional phenotype. The active repression of TCR signaling in the dysfunctional CD8^+^ T cells was further supported by the pathway analysis in which the expression of key components of the TCR signaling complex was coordinately downregulated in these tumors (58). The striking similarity between features that promote long-term survival (the present study), and those that predict response to checkpoint inhibitors (45) narrows the scope of definition of inflamed tumors to those that harbor functionally active, non-anergic and non-exhausted T cells. Further support to this hypothesis came from the fact that signature for functionally active CD8^+^ T cells were higher in melanoma patients who responded to Ipilimumab or Nivolumab monotherapies [Figure 5D, data from CA209-038 study 59].

**Figure 6:**
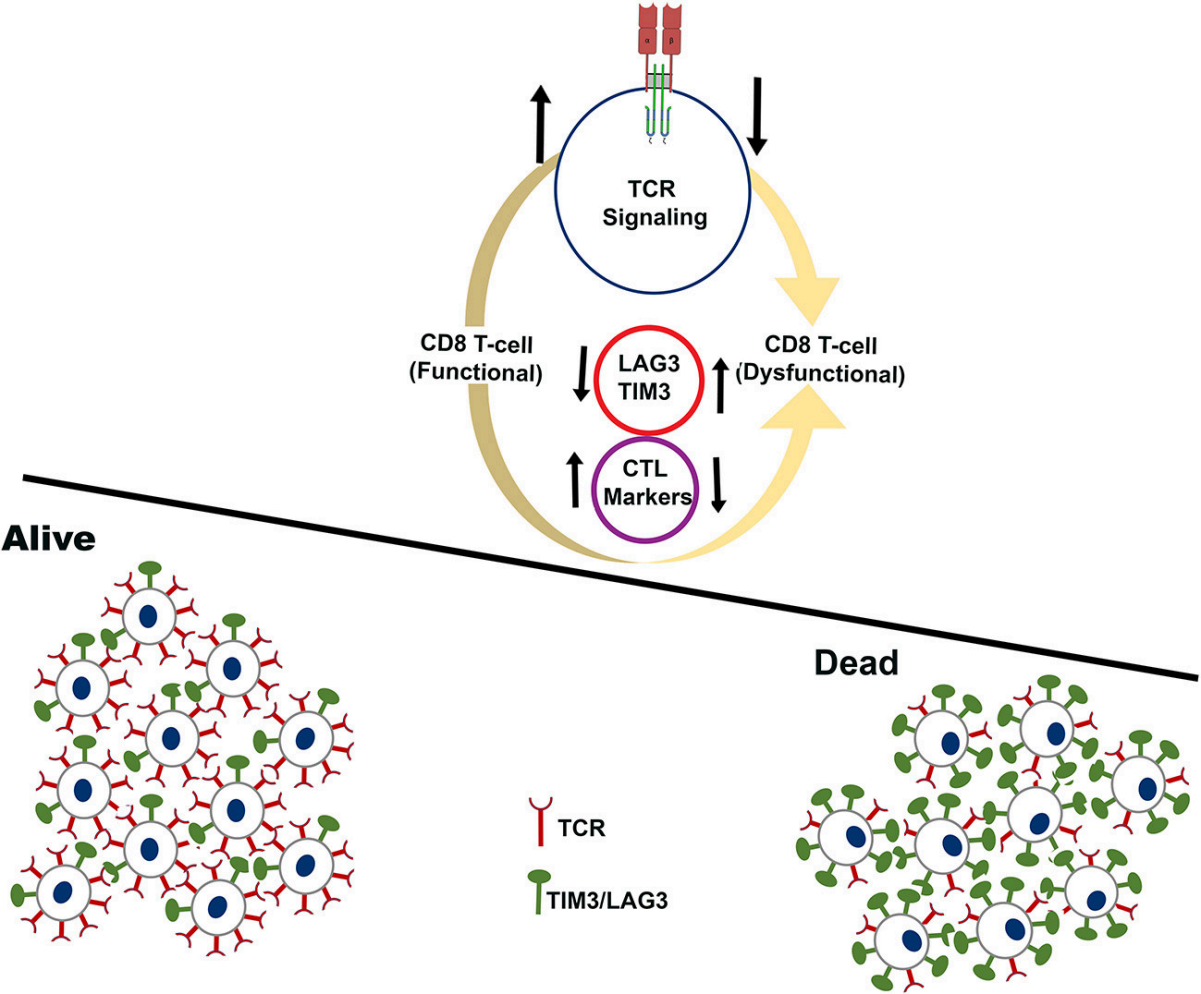
Schematic showing the immune microenvironment of tumors that experience long-term survival benefit (alive) over tumors that fail to show benefit (dead). The analysis is restricted to tumors that have high infiltration of CD8^+^ T-cells. Cartoon representation of tumors that experience long-term survival benefit are infiltrated by functional CD8^+^ T-cells characterized by higher expression of CTL markers, and higher expression of TCR signaling genes.

Taken together, the pan-cancer immune landscape analysis reveals critical determinants of long-term survival pointing to an integrated approach that can be designed for selecting patients who will benefit from cancer immunotherapy treatment as schematically represented in Figure 6. Our analysis uncovers key features of inflamed tumors that shift the balance from immunity to tolerance.

## Materials and Methods

### Creating Cell Type-Specific Gene Signatures

Unique gene expression signatures defining a specific immune cell type were identified by analyzing large microarray datasets (restricted to Affymetrix human genome U133 plus 2.0 platform) of pure immune cell types with the workflow described in Figure 1A. Genes with very high specificity for a given cell type were identified by calculating the average rank score of a given gene in each cell-type. The plasticity of the genes was assessed using the Marker Evaluation Score (MES) as described in Wang et al. (13). Genes were further refined by analyzing the median expression of the genes in pure RNA-Seq data (Supplementary Table 1) and further validated using multiple datasets described below.

### Determination of Cell-Type Specific Immune Scores

Immune scores were derived for each cell type in our analysis using the ssGSEA using the gene signatures discovered for each cell type (14). For a given sample, gene expression values were rank-normalized and rank-ordered. The goal of ssGSEA is to determine whether the members of gene signatures (S) are randomly distributed throughout all the protein-coding genes (L) or primarily found at the top or bottom. We calculate an enrichment score (ES) that reflects the degree to which a set S is over-represented at the extremes (top or bottom) of the entire ranked list of all the protein-coding genes (L). The score is calculated by walking down the list L, increasing a running-sum statistic when we encounter a gene in S and decreasing it when we encounter genes not in S. The scores are further normalized by dividing the score using the total number of protein-coding genes. For a randomly distributed S, ES(S) will be relatively small, but if it is enriched at the top or bottom of the list, or otherwise non-randomly distributed, then ES(S) will be correspondingly high.

### Validation of the MGESPs

RNA-Seq expression data was downloaded from the GEO database (60) for the studies GSE60424 (15), ERR431583 (61), and GSE100382 (16) (details of the data can be found in Supplementary Table 1). The FPKM values were used to generate the immune scores using ssGSEA based approach for each cell type in our analysis as well as signatures from other groups. The t-sne plot (18) and hierarchical clustering plot were generated using the immune scores for the same data-set using “Rtsne package.” The microarray expression raw data for the studies were downloaded from GEO and RMA normalized using “Affy package.” Batch effects were removed using “COMBAT package” and immune scores were calculated as described earlier. Microarray-based gene expression data and Flow cytometry data from the study SDY305 were downloaded from Immport database. RMA normalization was performed on the microarray data using “Affy package,” and median expression was used for multiple probes. Microarray and FACS paired data were pulled out using Subject Accession Ids. The samples lacking paired data were removed from the analysis. Pearson correlation analysis of the cell fractions with the immune scores was done using “corrplot package.” Single cell expression data (2,764 single cells) covering B cells, CD8, Macrophages, and NK cells (10) was downloaded from the GEO database (GSE72056) and immune scores were computed using ssGSEA based approach. The method can be accessed at https://oncopepttume.medgenome.com/.

### Pan-Cancer Analysis of TCGA Data

The pan-cancer level 3 RSEM normalized gene-level expression data for the TCGA samples and their annotations were downloaded from TCGA data portal (62) to calculate the scores for each immune cell type. The ssGSEA provided by R package ESTIMATE and protein-coding gene-level expression data was used to calculate the rank normalized scores for the assessment of infiltration levels of 8 different immune cell types including B-cell, CD4, CD8^+^, and T-regulatory as adaptive and monocyte, macrophage, NK, and neutrophil as innate immune cells in 9,640 tumor samples from 33 different tumor types defined by TCGA. Plots were generated using the ggplot package in R. To understand a more focused view of the immune infiltration we divided all the samples for each tumor type to four quartiles (Q1–Q4) based on each immune cell type score where Q1 represents high score samples for the given cell type. Next, we analyzed the fraction of tumor samples across the different cancer types present in each quartile described in Figure 2A, right panel. We further estimated the fraction of samples in each cancer across the different samples and represented it as a heatmap. Pearson correlation analysis for each cancer using the immune cell type-specific scores was done using “corrplot R package.”

The tumor stage data for 25 cancers were downloaded from TCGA. The pathological stage of the tumor was used to segregate the samples into the stages 1, 2, 3, and 4. For each cell type, the median immune score of the samples in each stage was used to generate the pie charts for all the 25 cancers. One-way ANOVA test was used to determine significant variations in immune scores across stages for each cell-type.

We further clustered all the 9,640 TCGA tumor samples using all the eight immune cell type-specific scores and we divided the samples into 4 major clusters. We looked for tumor content Epithelial, Immune and stromal using signatures described in Yoshihara et al. (43) and different immune cell infiltration for these 4 groups using our MGESPs. Hierarchical clustering was performed for normalized gene expression values of all the protein-coding genes (63). Euclidean distance and the complete linkage clustering method is used for hierarchical clustering. The analysis is performed using the R language and additional packages: ggplot2, reshape2, and ggrepel.

The REACTOME database (44) was used to analyze the pathways enriched using genes upregulated and downregulated with fold change values >2 or fold change values < 2 in each of these clusters. The significantly enriched pathways and genes from cluster 4 were further analyzed. Correlation of CD8^+^ expression with the expression of cell surface markers was calculated using Pearson correlation coefficient. *P*-values and confidence intervals for each pair of input features were calculated using the cor.mtest function in R. A significance cutoff of 0.95 confidence interval was used.

### Survival Analysis

Out of 9,640 tumor samples downloaded for 33 different tumor types, 9,548 samples had survival information and these samples were used for downstream analysis. For each immune cell type, top and bottom 20 percent samples were selected as high and low cell type-infiltrated groups for each tumor type separately. Further 23 tumors were selected based on minimum 30 samples in each high and low infiltration group. For these selected samples survival time in days and vital status was used for survival analysis between two groups. The two patient cohorts are compared by a Kaplan-Meier survival plot, and the hazard ratio with 95% confidence intervals and log-rank *P*-values are calculated. Survival analysis was performed and represented using R and additional package survival, ggplot2, reshape2, ggrepel, and ggfortify. Additionally, in order to understand the effect of two cell types on survival samples with high and low scores for both the cell types were used and survival analysis was performed for seven different cancers for which sufficient data were available (30 samples in each group).

### Cox Regression Analysis

T cell activation score was calculated as a weighted average of gene expression of the genes GRAP2, CD247, CD3E, CD3D, ZAP70, CD8B, CD1C, TDGF1, TCF7, SH2D1A, LCK, CTSL2 using the formula given below:

(1) $$\begin{array}{r} \frac{\sum \left(wi*X\right)}{\sum wi} \end{array}$$

where the weight (wi) is the reciprocal of variance of the expression level of a gene, and X is the value of gene expression (64). Multivariate Cox proportional hazards regression models were used to assess the associations of CD8 T-cell functionality in the high CD8 T cell clusters. T cell activation scores were treated as categorical variables in the models. Binary T cell activation scores, high or low, was defined based on the cutoff value of T cell activation scores determined by an algorithm of the maximization of hazard ratio adopted in several papers (65). Association analyses were adjusted for patient's age at diagnosis and disease stage and. Univariate and multivariate analysis was used to compute the Cox proportional hazard regression models. In all statistical analyses, a *p*-value < 0.05 was considered significant surminer, survival, and survMisc packages was used in R for the analysis.

## Author Contributions

AC conceived the project. MM, NM, and SP developed the OncoPeptTUME framework under the guidance of RoG, AC, and RaG. MM, NM, SP, and AP performed the analysis of TCGA data and validation of the signatures based on design by RaG and AC. LI implemented the web version of OncoPeptTUME. RoG provided critical inputs and reviewed the manuscript. MM, RaG, and AC wrote the manuscript. All authors read the manuscript and approved it.

### Conflict of Interest Statement

The authors are full-time employees of MedGenome.

## References

[1] Hanahan D Coussens LM. Accessories to the crime: functions of cells recruited to the tumor microenvironment. Cancer Cell (2012) 21:309–22. 10.1016/j.ccr.2012.02.022 22439926

[2] Quail D Joyce J. Microenvironmental regulation of tumor progression and metastasis. Nat Med. (2013) 19:1423–37. 10.1038/nm.3394 24202395 PMC3954707

[3] Joyce JA Fearon DT. T cell exclusion, immune privilege, and the tumor microenvironment. Science (2015) 348:74–80. 10.1126/science.aaa6204 25838376

[4] Hackl H Charoentong P Finotello F Trajanoski Z. Computational genomics tools for dissecting tumour–immune cell interactions. Nat Rev Genet. (2016) 17:441–58. 10.1038/nrg.2016.67 27376489

[5] Avila Cobos F Vandesompele J Mestdagh P De Preter K. Computational deconvolution of transcriptomics data from mixed cell populations. Bioinforma Oxf Engl. (2018) 34:1969–79. 10.1093/bioinformatics/bty019 29351586

[6] Rooney MS Shukla SA Wu CJ Getz G Hacohen N. Molecular and genetic properties of tumors associated with local immune cytolytic activity. Cell (2015) 160:48–61. 10.1016/j.cell.2014.12.033 25594174 PMC4856474

[7] Bindea G Mlecnik B Tosolini M Kirilovsky A Waldner M Obenauf AC. Spatiotemporal dynamics of intratumoral immune cells reveal the immune landscape in human cancer. Immunity (2013) 39:782–95. 10.1016/j.immuni.2013.10.003 24138885

[8] Charoentong P Finotello F Angelova M Mayer C Efremova M Rieder D. Pan-cancer immunogenomic analyses reveal genotype-immunophenotype relationships and predictors of response to checkpoint blockade. Cell Rep. (2017) 18:248–62. 10.1016/j.celrep.2016.12.019 28052254

[9] Aran D Hu Z Butte AJ. xCell: digitally portraying the tissue cellular heterogeneity landscape. Genome Biol. (2017) 18:220. 10.1186/s13059-017-1349-1 29141660 PMC5688663

[10] Tirosh I Izar B Prakadan SM Wadsworth MH Treacy D Trombetta JJ. Dissecting the multicellular ecosystem of metastatic melanoma by single-cell RNA-seq. Science (2016) 352:189–96. 10.1126/science.aad0501 27124452 PMC4944528

[11] Zheng C Zheng L Yoo J-K Guo H Zhang Y Guo X. Landscape of infiltrating T cells in liver cancer revealed by single-cell sequencing. Cell (2017) 169:1342–56.e16. 10.1016/j.cell.2017.05.035 28622514

[12] Venteicher AS Tirosh I Hebert C Yizhak K Neftel C Filbin MG. Decoupling genetics, lineages, and microenvironment in IDH-mutant gliomas by single-cell RNA-seq. Science (2017) 355:8478. 10.1126/science.aai8478 28360267 PMC5519096

[13] Wang P Yang Y Han W Ma D. ImmuSort, a database on gene plasticity and electronic sorting for immune cells. Sci Rep. (2015) 5:10370. 10.1038/srep10370 25988315 PMC4437374

[14] Subramanian A Tamayo P Mootha VK Mukherjee S Ebert BL Gillette MA. Gene set enrichment analysis: a knowledge-based approach for interpreting genome-wide expression profiles. Proc Natl Acad Sci USA. (2005) 102:15545–50. 10.1073/pnas.0506580102 16199517 PMC1239896

[15] Linsley PS Speake C Whalen E Chaussabel D. Copy number loss of the interferon gene cluster in melanomas is linked to reduced T cell infiltrate and poor patient prognosis. PLoS ONE (2014) 9:e109760. 10.1371/journal.pone.0109760 25314013 PMC4196925

[16] Park SH Kang K Giannopoulou E Qiao Y Kang K Kim G. Type I interferons and the cytokine TNF cooperatively reprogram the macrophage epigenome to promote inflammatory activation. Nat Immunol. (2017) 18:1104–16. 10.1038/ni.3818 28825701 PMC5605457

[17] Bhattacharya S Andorf S Gomes L Dunn P Schaefer H Pontius J. ImmPort: disseminating data to the public for the future of immunology. Immunol Res. (2014) 58:234–9. 10.1007/s12026-014-8516-1 24791905

[18] Li W Cerise JE Yang Y Han H. Application of t-SNE to human genetic data. J Bioinform Comput Biol. (2017) 15:1750017. 10.1142/S0219720017500172 28718343

[19] Liu KG Gupta S Goel S. Immunotherapy: incorporation in the evolving paradigm of renal cancer management and future prospects. Oncotarget (2017) 8:17313–27. 10.18632/oncotarget.14388 28061473 PMC5370043

[20] Geissler K Fornara P Lautenschläger C Holzhausen H-J Seliger B Riemann D. Immune signature of tumor infiltrating immune cells in renal cancer. Oncoimmunology (2015) 4:e985082. 10.4161/2162402X.2014.985082 25949868 PMC4368143

[21] Melero I Rouzaut A Motz GT Coukos G. T-cell and NK-cell infiltration into solid tumors: a key limiting factor for efficacious cancer immunotherapy. Cancer Discov. (2014) 4:522–6. 10.1158/2159-8290.CD-13-0985 24795012 PMC4142435

[22] Steinhart Z Pavlovic Z Chandrashekhar M Hart T Wang X Zhang X Genome-wide CRISPR screens reveal a Wnt-FZD5 signaling circuit as a druggable vulnerability of RNF43-mutant pancreatic tumors. Nat Med. (2017) 23:60–8. 10.1038/nm.4219 27869803

[23] Cui H-Y Wang S-J Miao J-Y Fu Z-G Feng F Wu J. CD147 regulates cancer migration via direct interaction with Annexin A2 and DOCK3-β-catenin-WAVE2 signaling. Oncotarget (2015) 7:5613–29. 10.18632/oncotarget.6723 26716413 PMC4868709

[24] Tesniere A Zitvogel L Kroemer G. The immune system: taming and unleashing cancer. Discov Med. (2006) 6:211–6. 17250785

[25] Colotta F Allavena P Sica A Garlanda C Mantovani A. Cancer-related inflammation, the seventh hallmark of cancer: links to genetic instability. Carcinogenesis (2009) 30:1073–81. 10.1093/carcin/bgp127 19468060

[26] Huh JW Lee JH Kim HR. Prognostic significance of tumor-infiltrating lymphocytes for patients with colorectal cancer. Arch Surg Chic Ill 1960 (2012) 147:366–72. 10.1001/archsurg.2012.35 22508783

[27] Adams SF Levine DA Cadungog MG Hammond R Facciabene A Olvera N. Intraepithelial T cells and tumor proliferation: impact on the benefit from surgical cytoreduction in advanced serous ovarian cancer. Cancer (2009) 115:2891–902. 10.1002/cncr.24317 19472394 PMC2754811

[28] Mao Y Qu Q Chen X Huang O Wu J Shen K. The prognostic value of tumor-infiltrating lymphocytes in breast cancer: a systematic review and meta-analysis. PLoS ONE (2016) 11:e0152500 10.1371/journal.pone.0152500 27073890 PMC4830515

[29] Badalamenti G Fanale D Incorvaia L Barraco N Listì A Maragliano R. Role of tumor-infiltrating lymphocytes in patients with solid tumors: Can a drop dig a stone? Cell Immunol. (2018) S0008–8749(18)30014–5. 10.1016/j.cellimm.2018.01.013 29395859

[30] The Immune Contexture in Cancer Prognosis and Treatment - PubMed - NCBI. Available online at: https://www.ncbi.nlm.nih.gov/pubmed/?term=28741618 (Accessed April 2, 2018).

[31] Sharma P Shen Y Wen S Yamada S Jungbluth AA Gnjatic S. CD8 tumor-infiltrating lymphocytes are predictive of survival in muscle-invasive urothelial carcinoma. Proc Natl Acad Sci USA. (2007) 104:3967–72. 10.1073/pnas.0611618104 17360461 PMC1820692

[32] Schumacher K Haensch W Röefzaad C Schlag PM. Prognostic significance of activated CD8^+^ T cell infiltrations within esophageal carcinomas. Cancer Res. (2001) 61:3932–6. Available online at: http://cancerres.aacrjournals.org/content/61/10/3932.long 11358808

[33] Sorbye SW Kilvaer T Valkov A Donnem T Smeland E Al-Shibli K. Prognostic Impact of Lymphocytes in Soft Tissue Sarcomas. PLOS ONE (2011) 6:e14611. 10.1371/journal.pone.0014611 21298041 PMC3029277

[34] Hadrup S Donia M Thor Straten P. Effector CD4 and CD8 T cells and their role in the tumor microenvironment. Cancer Microenviron. (2012) 6:123–33. 10.1007/s12307-012-0127-6 23242673 PMC3717059

[35] Moreno-Cubero E Larrubia J-R. Specific CD8^+^ T cell response immunotherapy for hepatocellular carcinoma and viral hepatitis. World J Gastroenterol. (2016) 22:6469–83. 10.3748/wjg.v22.i28.6469 27605882 PMC4968127

[36] Bastman JJ Serracino HS Zhu Y Koenig MR Mateescu V Sams SB. Tumor-infiltrating T cells and the PD-1 checkpoint pathway in advanced differentiated and anaplastic thyroid cancer. J Clin Endocrinol Metab. (2016) 101:2863–73. 10.1210/jc.2015-4227 27045886 PMC4929840

[37] Pretscher D Distel LV Grabenbauer GG Wittlinger M Buettner M Niedobitek G. Distribution of immune cells in head and neck cancer: CD8^+^ T-cells and CD20^+^B-cells in metastatic lymph nodes are associated with favourable outcome in patients with oro- and hypopharyngeal carcinoma. BMC Cancer (2009) 9:292. 10.1186/1471-2407-9-292 19698134 PMC2739224

[38] Chen DS Mellman I. Elements of cancer immunity and the cancer-immune set point. Nature (2017) 541:321–30. 10.1038/nature21349 28102259

[39] Aran D Lasry A Zinger A Biton M Pikarsky E Hellman A. Widespread parainflammation in human cancer. Genome Biol. (2016) 17:145. 10.1186/s13059-016-0995-z 27386949 PMC4937599

[40] Varn FS Wang Y Mullins DW Fiering S Cheng C. Systematic pan-cancer analysis reveals immune cell interactions in the tumor microenvironment. Cancer Res. (2017) 77:1271–82. 10.1158/0008-5472.CAN-16-2490 28126714 PMC5798883

[41] Li B Severson E Pignon J-C Zhao H Li T Novak J. Comprehensive analyses of tumor immunity: implications for cancer immunotherapy. Genome Biol. (2016) 17:174. 10.1186/s13059-016-1028-7 27549193 PMC4993001

[42] Becht E Giraldo NA Lacroix L Buttard B Elarouci N Petitprez F Estimating the population abundance of tissue-infiltrating immune and stromal cell populations using gene expression. Genome Biol. (2016) 17:5 10.1186/s13059-016-1070-5 27908289 PMC5134277

[43] Yoshihara K Shahmoradgoli M Martínez E Vegesna R Kim H Torres-Garcia W. Inferring tumour purity and stromal and immune cell admixture from expression data. Nat Commun. (2013) 4:3612. 10.1038/ncomms3612 24113773 PMC3826632

[44] Fabregat A Jupe S Matthews L Sidiropoulos K Gillespie M Garapati P. The reactome pathway knowledgebase. Nucleic Acids Res. (2018) 46:D649–55. 10.1093/nar/gkx1132 29145629 PMC5753187

[45] Riaz N Havel JJ Makarov V Desrichard A Urba WJ Sims JS. Tumor and microenvironment evolution during immunotherapy with Nivolumab. Cell (2017) 171:934–49.e15. 10.1016/j.cell.2017.09.028 29033130 PMC5685550

[46] Callahan MK Postow MA Wolchok JD. CTLA-4 and PD-1 pathway blockade: combinations in the clinic. Front Oncol. (2014) 4:385. 10.3389/fonc.2014.00385 25642417 PMC4295550

[47] Khalil DN Smith EL Brentjens RJ Wolchok JD The future of cancer treatment: immunomodulation, CARs and combination immunotherapy. Nat Rev Clin Oncol. (2016) 13:394 10.1038/nrclinonc.2016.65 27118494 PMC5558237

[48] Wolchok JD Chiarion-Sileni V Gonzalez R Rutkowski P Grob J-J Cowey CL. Overall survival with combined nivolumab and ipilimumab in advanced melanoma. N Engl J Med. (2017) 377:1345–56. 10.1056/NEJMoa1709684 28889792 PMC5706778

[49] Taube JM Klein A Brahmer JR Xu H Pan X Kim JH. Association of PD-1, PD-1 ligands, and other features of the tumor immune microenvironment with response to anti-PD-1 therapy. Clin Cancer Res Off J Am Assoc Cancer Res. (2014) 20:5064–74. 10.1158/1078-0432.CCR-13-3271 24714771 PMC4185001

[50] Rizvi NA Hellmann MD Snyder A Kvistborg P Makarov V Havel JJ. Cancer immunology. mutational landscape determines sensitivity to PD-1 blockade in non-small cell lung cancer. Science (2015) 348:124–8. 10.1126/science.aaa1348 25765070 PMC4993154

[51] Chen P-L Roh W Reuben A Cooper ZA Spencer CN Prieto PA. Analysis of immune signatures in longitudinal tumor samples yields insight into biomarkers of response and mechanisms of resistance to immune checkpoint blockade. Cancer Discov. (2016) 6:827–37. 10.1158/2159-8290.CD-15-1545 27301722 PMC5082984

[52] Mikucki ME Fisher DT Matsuzaki J Skitzki JJ Gaulin NB Muhitch JB. Non-redundant requirement for CXCR3 signalling during tumoricidal T-cell trafficking across tumour vascular checkpoints. Nat Commun. (2015) 6:7458. 10.1038/ncomms8458 26109379 PMC4605273

[53] Tan LY Martini C Fridlender ZG Bonder CS Brown MP Ebert LM. Control of immune cell entry through the tumour vasculature: a missing link in optimising melanoma immunotherapy? Clin Transl Immunol. (2017) 6:e134. 10.1038/cti.2017.7 28435677 PMC5382436

[54] Jiang X Hao H-X Growney JD Woolfenden S Bottiglio C Ng N. Inactivating mutations of RNF43 confer Wnt dependency in pancreatic ductal adenocarcinoma. Proc Natl Acad Sci USA. (2013) 110:12649–54. 10.1073/pnas.1307218110 23847203 PMC3732970

[55] Koo B-K Spit M Jordens I Low TY Stange DE van de Wetering M. Tumour suppressor RNF43 is a stem-cell E3 ligase that induces endocytosis of Wnt receptors. Nature (2012) 488:665–9. 10.1038/nature11308 22895187

[56] Spranger S Bao R Gajewski TF. Melanoma-intrinsic β-catenin signalling prevents anti-tumour immunity. Nature (2015) 523:231–5. 10.1038/nature14404 25970248

[57] Crespo J Sun H Welling TH Tian Z Zou W. T cell anergy, exhaustion, senescence and stemness in the tumor microenvironment. Curr Opin Immunol. (2013) 25:214–21. 10.1016/j.coi.2012.12.003 23298609 PMC3636159

[58] Chappert P Schwartz RH. Induction of T cell anergy: integration of environmental cues and infectious tolerance. Curr Opin Immunol. (2010) 22:552–9. 10.1016/j.coi.2010.08.005 20869863 PMC2981408

[59] Choueiri TK Fishman MN Escudier B McDermott DF Drake CG Kluger H. Immunomodulatory activity of nivolumab in metastatic renal cell carcinoma. Clin Cancer Res Off J Am Assoc Cancer Res. (2016) 22:5461–71. 10.1158/1078-0432.CCR-15-2839 27169994 PMC5106340

[60] Gene Expression Omnibus (GEO) - The NCBI Handbook - NCBI Bookshelf Available online at: https://www.ncbi.nlm.nih.gov/books/NBK159736/ (Accessed March 4, 2018).

[61] Home - SRA - NCBI Available online at: https://www.ncbi.nlm.nih.gov/sra (Accessed March 4, 2018).

[62] The Cancer Genome Atlas Home Page Cancer Genome Atlas - Natl Cancer Inst Available online at: https://cancergenome.nih.gov/ (Accessed March 4, 2018).

[63] Müllner D Modern Hierarchical, Agglomerative Clustering Algorithms. ArXiv11092378 Cs Stat (2011). Available online at: http://arxiv.org/abs/1109.2378 (Accessed March 4, 2018).

[64] Lu L Bai Y Wang Z. Elevated T cell activation score is associated with improved survival of breast cancer. Breast Cancer Res Treat. (2017) 164:689–96. 10.1007/s10549-017-4281-x 28488141

[65] Contal C O'Quigley J An application of changepoint methods in studying the effect of age on survival in breast cancer. Comput Stat Data Anal. (1999) 30:253–70. 10.1016/S0167-9473(98)00096-6

