# Supplementary material for: A Computational Approach Identifies Immunogenic Features of Prognosis in Human Cancers
Source: Front Immunol. 2018 Dec 21;9:3017. doi: 10.3389/fimmu.2018.03017 (PMC6308325; doi:10.3389/fimmu.2018.03017)
Supplement: Supplementary file 1 [file Data_Sheet_1.docx]

**A Computational Approach Identifies Immunogenic Features of Prognosis**

**in Human Cancers**

Malini Manoharan^1†^, Nitin Mandloi ^1†^, Sushri Priyadarshini ^1^, Ashwini Patil ^2^, Rohit Gupta^1^, Laxman Iyer ^1^, Ravi Gupta^1^* and Amitabha Chaudhuri^2^*

^1^MedGenome Labs Ltd., Bangalore, India, ^2^MedGenome Inc. Foster City, CA, United States

**
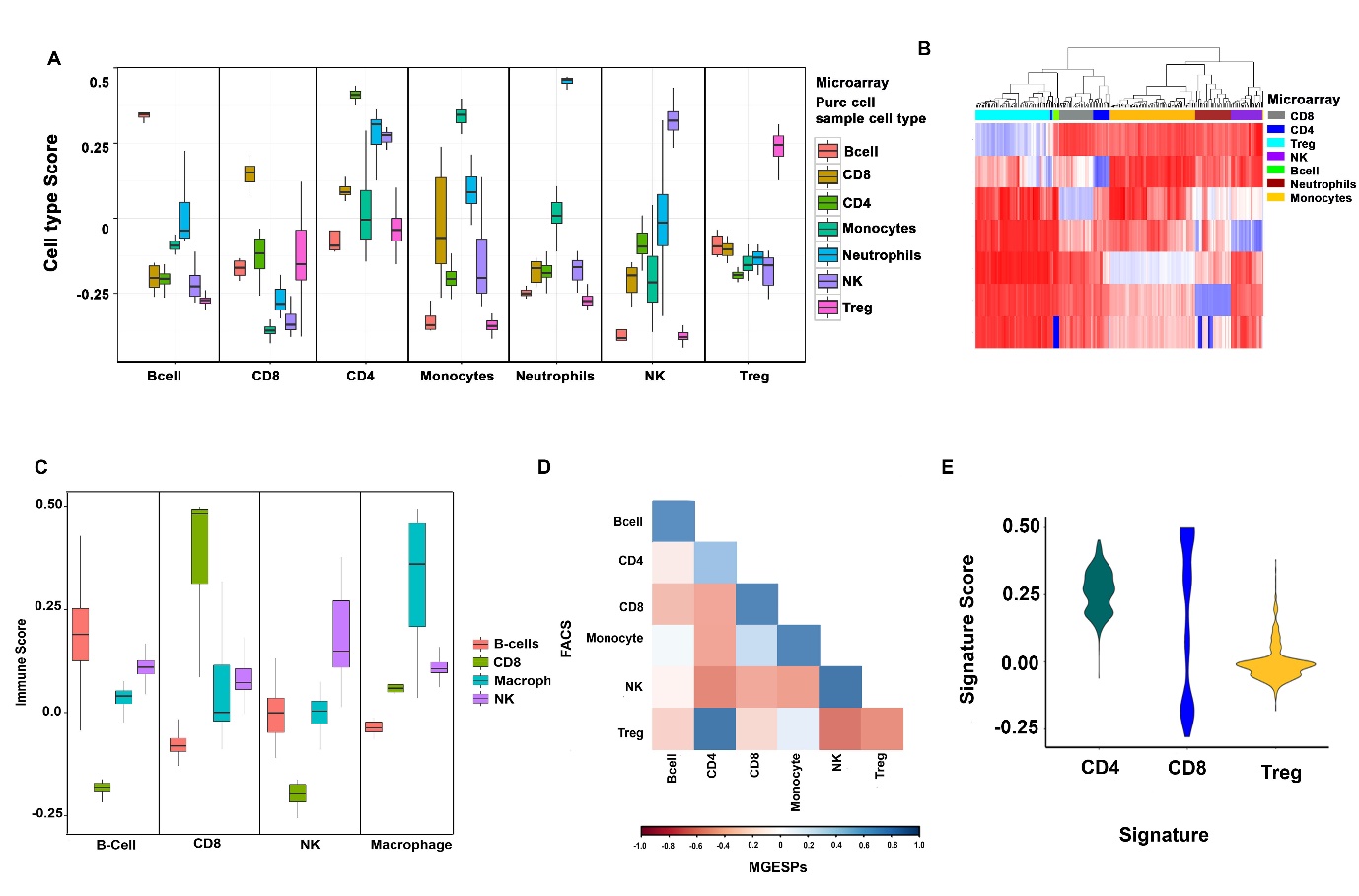
**

**Supplementary Figure 1. Validation of minimal gene expression signature profiles (MGESPs) using microarray data. A.** Validation of MGESPs on Microarray data represented as boxplots with signature on the x-axis and immune score on the y-axis. Each facet represents the immune score calculated for the signature mentioned on the x-axis for both their cognate and non-cognate cell types. A higher score was obtained for the cognate cell types as represented in each facet. **B.** Hierarchical clustering of immune cell-types on RNA-Seq data from pure immune cells. **C.** Represents a box plot with signature on the x-axis and immune score on the y-axis as described in (**A**) highlighting the segregation of immune cells by MGESPs from single-cell RNA-Seq data. **D.** Comparison of MGESPs with FACS data. With signature on the x-axis and immune cell fraction on the y-axis the figure represents the correlation of the immune scores with the cell fractions obtained using FACS data. **E.** CD4, CD8 and Treg scores derived by applying signatures of different T cells on single-cell sequence data on a group of cells labeled as T cells.

**Supplementary Figure 2. The landscape of Immune cell infiltration across different cancers from the TCGA data.** Distribution of immune infiltration scores across 32 cancers. The dark line represents the median infiltration score. The number of samples in each cancer is represented as a bar chart on the top.


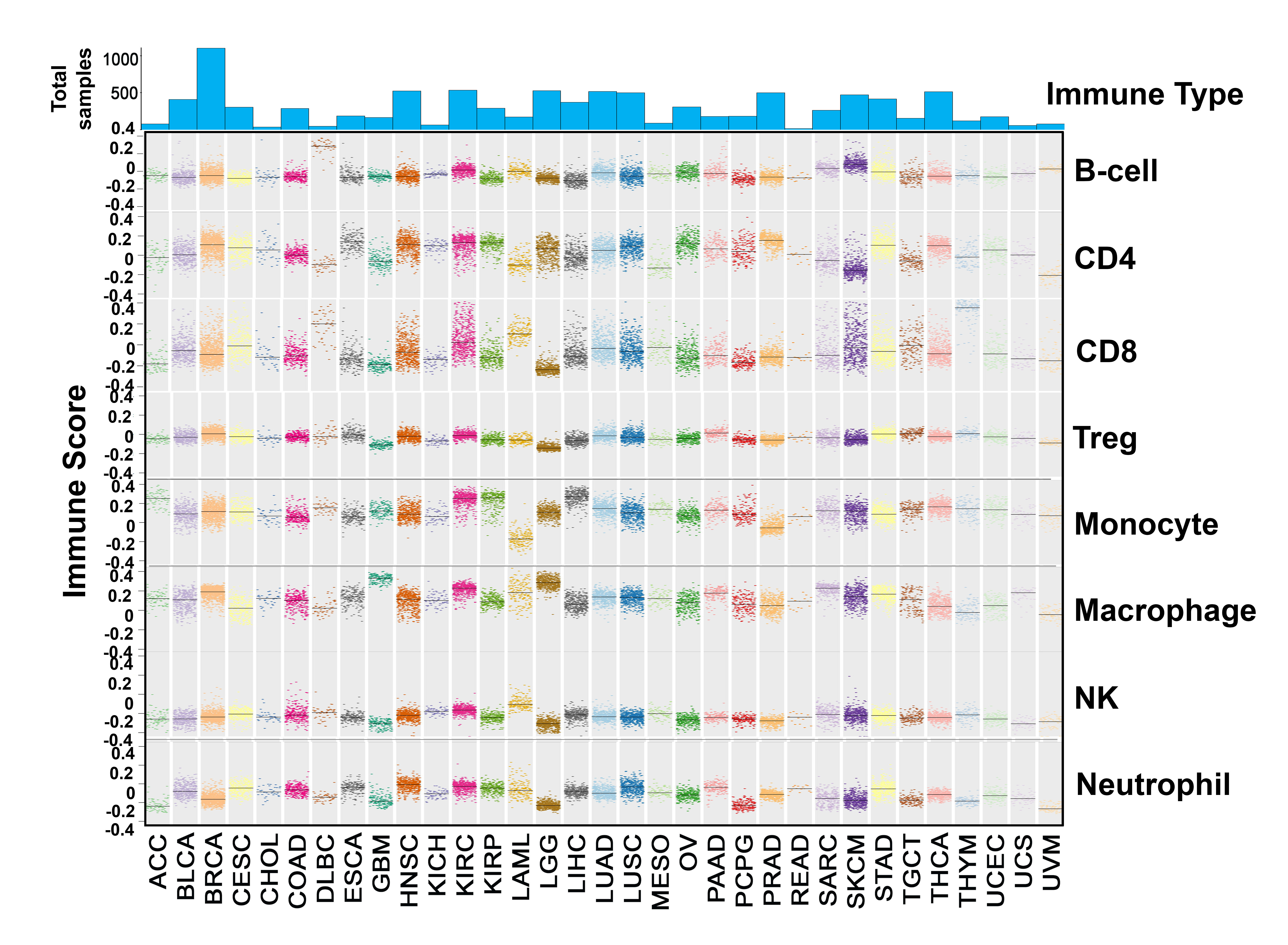


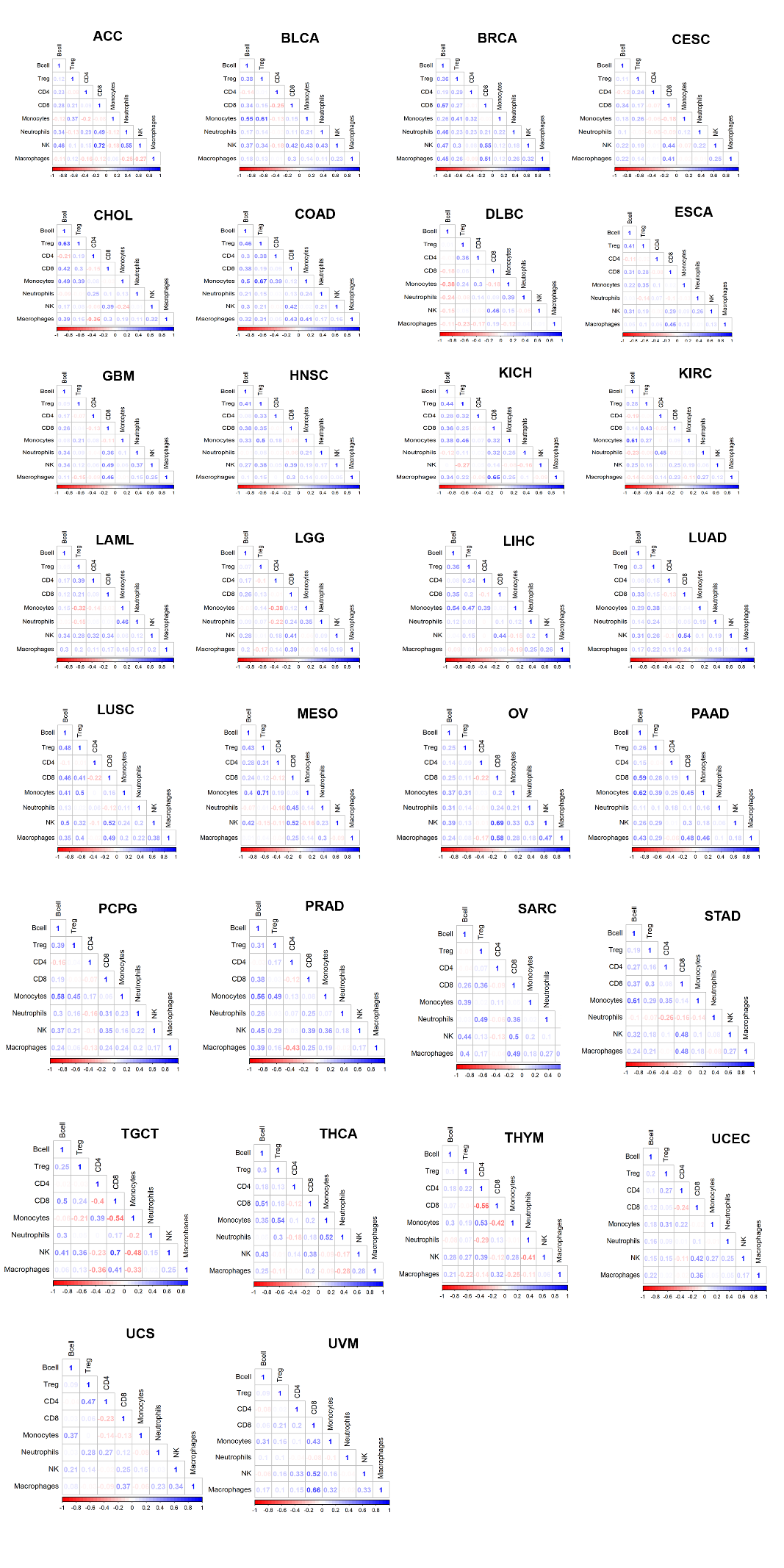


**Supplementary Figure 3. Correlation of immune cell infiltration across cancers** Immune scores for each cell type was calculated for all the samples in the TCGA, and correlation of infiltration was calculated for all the cancers with more than 50 samples.


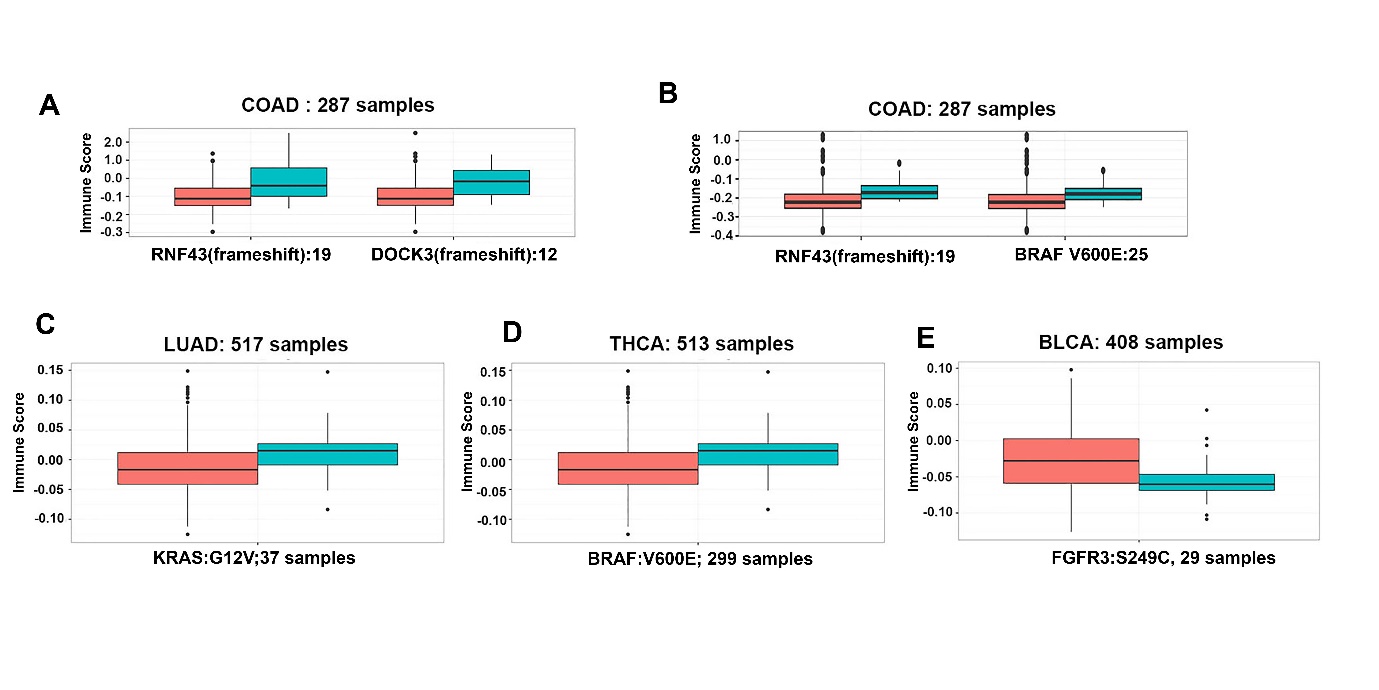


**Supplementary Figure 4. Enrichment of specific immune cells in tumors carrying driver mutations in oncogenes and tumor suppressor genes which impact CD8+ infiltration.** Tumors were selected for specific mutations in genes and profiled for immune infiltration using MGESPs. For each cancer type, tumors carrying specific gene mutations (blue) are compared with tumors lacking the same mutation (red) for enrichment or depletion of specific immune cell types. Enrichments or depletions with p <0.01 are shown in Table S3. Few examples from the list are shown in the figure. **A-B** Enrichment of mutations in RNF43 and DOCK-3 genes associated with higher infiltration of CD8^+^ T cells in colon adenocarcinoma (COAD) samples (represented in blue). **C.** Enrichment of KRAS G12V associated with higher infiltration of Treg cells in lung adenocarcinoma (LUAD) samples (represented in blue). **D.** Enrichment of BRAF V600E associated with higher infiltration of Treg cells in lung adenocarcinoma (THCA) samples (represented in blue). **E.** Depletion of FGFR3 S249C associated with higher infiltration of Treg cells in lung adenocarcinoma (THCA) samples (represented in blue).


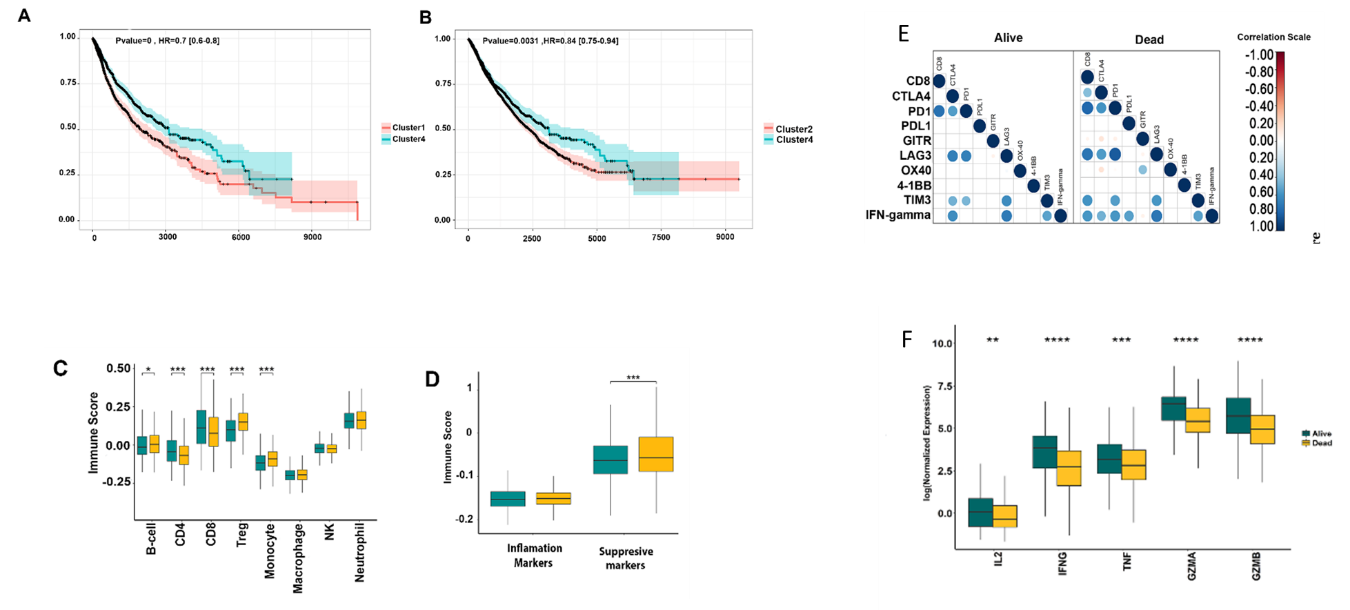


**Supplementary Figure 5. Factors affecting prognosis in the CD8^+^ T-cellhi cluster (cluster-4). A.** Survival differences between samples present in cluster-1 and cluster-4. **B.** Survival differences between samples present in cluster-2 and cluster-4. **C.** Immune landscape of tumors in alive and deceased groups from cluster-4. **D.** Boxplot showing the difference in scores between the alive and deceased groups using genes that describe inflammatory and immunosuppressive features. E. Correlation between CD8^+^ T cells and the expression of T cell activation/exhaustion markers. **F.** Boxplot showing significant change in expression of cytolytic markers between the alive and deceased groups (Wilcox text, P-value <0.05).

**
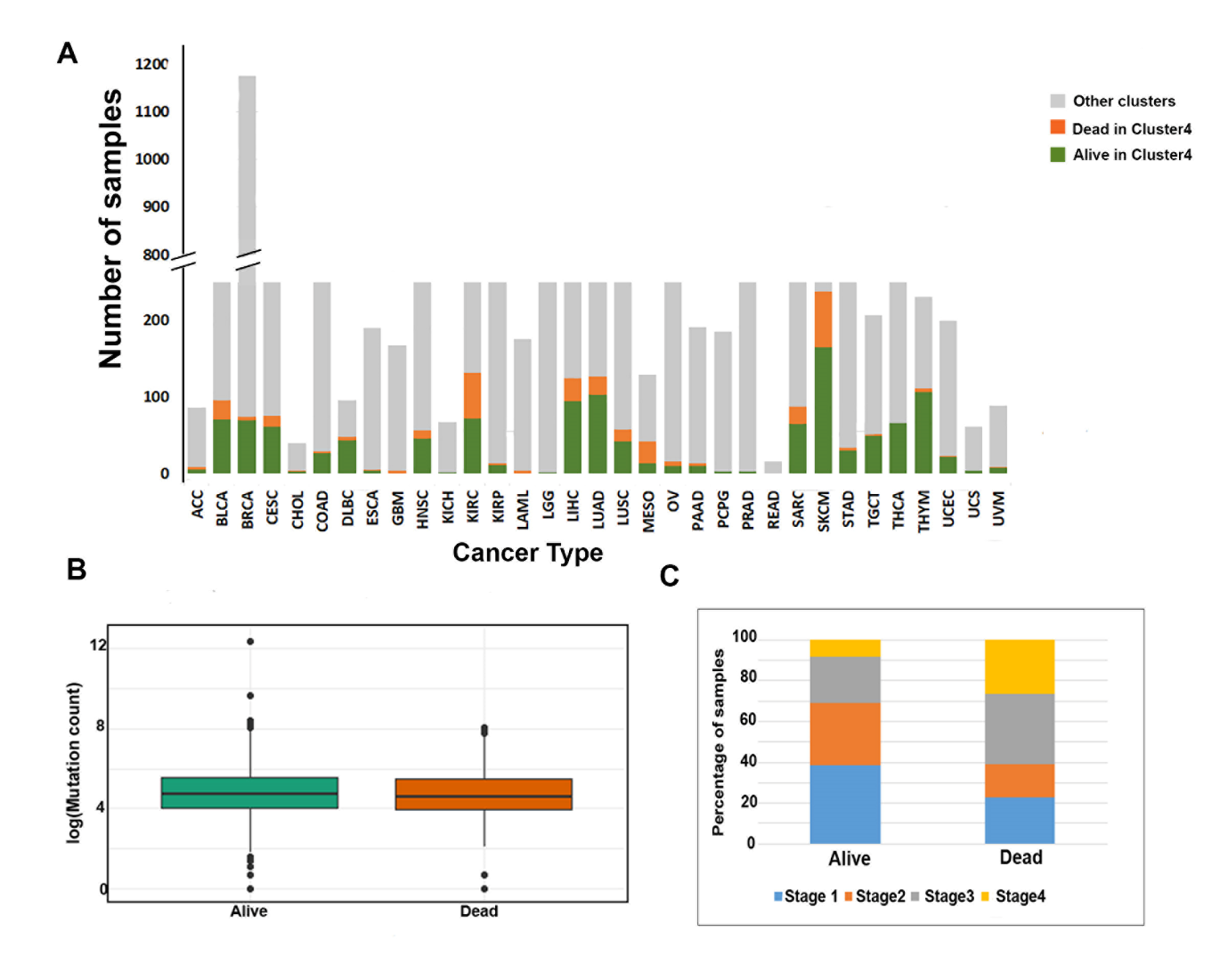
**

**Supplementary Figure 6. Analysis of other factors affecting survival in cluster-4 tumors. A)** Distribution of tumors belonging to the alive and dead groups across different cancers. Total number of samples and their distribution in Cluster-4 (CD8-high cluster). Alive (Green) and Dead (Orange) groups corresponding to each cancer are shown. The sample count for the remaining clusters (1-3) is shown as a Grey bar. B) Number of non-syn mutations in tumors belonging to the alive and the dead group of samples from Cluster-4. C) Distribution of early and late stage tumors in alive and dead groups.


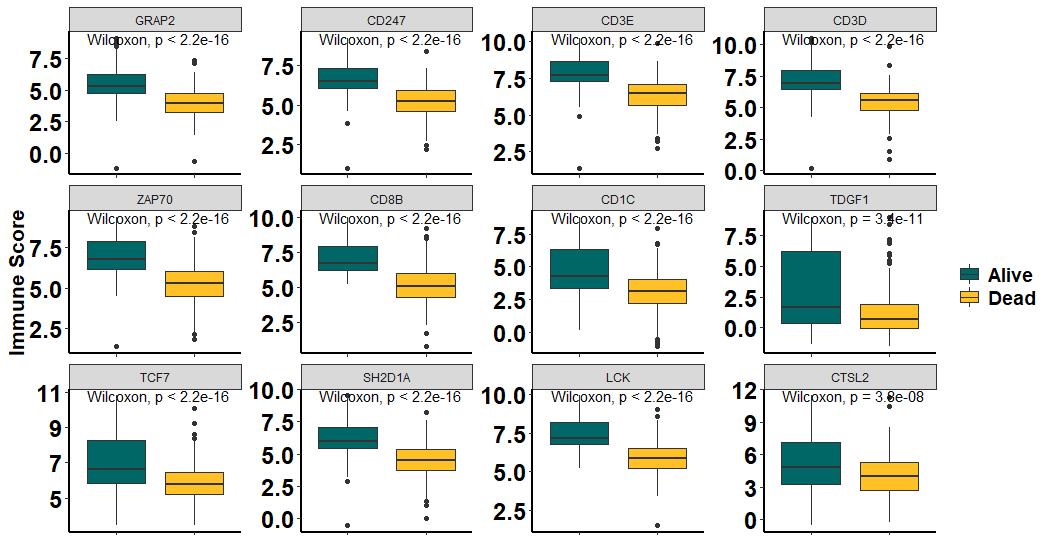


**Supplementary Figure 7. Differentially expressed TCR signalling genes in the alive and the deceased groups.** Normalized expression of the TCR signalling genes in the two groups from Cluster 4. (Wilcox text, P-value <0.05)
